# The antioxidant barrier, oxidative/nitrosative stress, and protein glycation in allergy: from basic research to clinical practice

**DOI:** 10.3389/fimmu.2024.1440313

**Published:** 2024-12-05

**Authors:** Grzegorz Biedrzycki, Blanka Wolszczak-Biedrzycka, Justyna Dorf, Mateusz Maciejczyk

**Affiliations:** ^1^ Hospital Pharmacy, Provincial Psychiatric Hospital, Olsztyn, Poland; ^2^ Department of Psychology and Sociology of Health and Public Health, University of Warmia and Mazury, Olsztyn, Poland; ^3^ Department of Clinical Laboratory Diagnostics, Medical University of Bialystok, Bialystok, Poland; ^4^ Department of Hygiene, Epidemiology and Ergonomics, Medical University of Bialystok, Bialystok, Poland

**Keywords:** allergy, oxidative stress, nitrosative stress, protein glycation, antioxidants

## Abstract

Recent studies indicate that oxidative/nitrosative stress is involved in the pathogenesis of asthma, allergic rhinitis, atopic dermatitis, and urticaria. The article aimed to review the latest literature on disruptions in redox homeostasis and protein glycation in allergy patients. It has been shown that enzymatic and non-enzymatic antioxidant systems are impaired in allergic conditions, which increases cell susceptibility to oxidative damage. Reactive oxygen/nitrogen species exacerbate the severity of asthma symptoms by activating inflammatory mediators that cause airway smooth muscle contraction, promote mucus hypersecretion, increase the permeability of lung capillaries, and damage cell membranes. Redox biomarkers could have considerable diagnostic potential in allergy patients. There is no compelling evidence to indicate that antioxidants reduce allergy symptoms’ severity or slow disease progression.

## Introduction

The pathogenesis of allergies has been studied extensively by researchers and clinicians for more than 100 years. The term “allergy” was first used in 1906 by Clemens von Pirquet to describe patients who were hyperresponsive or hypersensitive to exogenous substances (1). In the 1960s, Gell and Coombs defined allergy as an exaggerated or inappropriate immune response, and they divided allergic responses into four pathophysiological types: anaphylaxis (type I), antibody-mediated cytotoxic reactions (type II), immune complex-mediated reactions (type III), and cell-mediated delayed hypersensitivity (type IV) (2). Most atopic diseases (the Greek word *atopos* means “out of place”) are caused by type I hypersensitivity reactions. Anaphylaxis is induced antigen cross-linking of antigen-specific IgE antibodies that bind to high-affinity IgE receptors on the surface of basophils and mast cells. The main clinical manifestations of atopy are allergic rhinitis (AR), atopic dermatitis (AD), allergic (atopic) asthma and some allergies (3).

Allergies are increasingly prevalent in highly developed countries. According to estimates, atopic diseases affect 25-30% of the global population. Similarly to hypertension, coronary artery disease, and cancer, allergies have been classified as lifestyle diseases (4). Environmental pollution, low levels of physical activity, poor nutrition, and prolonged stress increase the risk of lifestyle diseases and contribute to the overproduction of reactive oxygen species (ROS). ROS are the by-products of normal metabolic processes in aerobic organisms. When present in physiological concentrations, they participate in the regulation of immune processes, including T cell activation and leukocyte adhesion to endothelial cells. However, environmental factors can increase ROS production (5). When more ROS are produced than scavenged, the resulting pro-oxidant/antioxidant imbalance leads to oxidative stress (6). A pro-oxidant/antioxidant imbalance can occur inside individual cells or in subcellular compartments without modifying the redox status of the cell or the entire organism (7). It should also be noted that lungs and skin are particularly sensitive to oxidative damage. These organs are directly exposed to high oxygen concentrations as well as oxidative stress caused by normal aerobic metabolism or exposure to irritant compounds and other xenobiotics in air (such as cigarette smoke and air pollutants) (8). To counteract the overproduction of ROS, the body has developed specialized antioxidant systems, whose first line of defense are antioxidant enzymes such as superoxide dismutase (SOD), catalase (CAT), glutathione peroxidase (GPx), but also other redox proteins such as thioredoxins (TRX), peroxiredoxins (PRX) and glutaredoxins. Non-enzymatic low-molecular-weight antioxidants (LMWA) include hydrophilic uric acid (UA), vitamin C and reduced glutathione (GSH; L-γ-glutamyl-L-cysteinyl-L-glycine) as well as lipophilic compounds like vitamins A, D and E (9, 10).

Numerous epidemiological and clinical studies have shown that oxidative stress directly or indirectly contributes to the risk of lifestyle diseases (11–14). Due to the decreased cellular proliferation, loss of adaptive immune function, and immunosenescence, ROS overproduction is an important hallmark of age-related diseases (15). Oxidative stress can exacerbate pre-existing health conditions, and it is one of the main causes of chronic inflammation. ROS are mainly produced in host defense response cells such as neutrophils and polymorphonuclear neutrophils (PMNs) (16). Allergens, chemicals and infections increase the ROS production, which activates nuclear factor kappa B (NF-KB) (17). Pro-inflammatory enzymes such as myeloperoxidase (MPO), NADPH oxidase (NOX), and inducible nitric oxide synthase (iNOS) are activated through cyclooxygenase and lipoxygenase pathways of arachidonic acid metabolism (5). These enzymes, in combination with NF-KB induction, not only increase the synthesis and secretion of cytokines, chemokines, and growth factors. They also lead to the overproduction of ROS and reactive nitrogen species (RNS) (18). Under these conditions, superoxide anion radical is produced in very large quantities. Superoxide can rapidly combine with nitric oxide (NO) to form peroxynitrite (ONOO^-^), the strongest oxidizing agent *in vivo (* 19). This reaction is three to four times faster than superoxide dismutation by SOD (18). RNS, in turn, induce nitrosative stress, adding to the pro-inflammatory burden of ROS (20). It was shown that oxidative stress is not only responsible for impaired intracellular signaling, but also abnormal immune responses and oxidative DNA damage (21). The damaging effects of inflammation-related oxidative stress are particularly exacerbated under hypoxic conditions. Low oxygen levels trigger a number of responses that are associated with numerous physiological and pathophysiological scenarios (22). Hypoxia results in inefficient respiratory electron transfer, leading to overproduction of mitochondrial ROS and irreversible intracellular damage (23, 24).

According to recent research, oxidative and nitrosative stress plays a key role in the pathogenesis of respiratory (including asthma) and skin allergies (AD, urticaria) (25–27). Moreover, food allergies were found to be associated with carbonyl stress resulting from the accumulation of advanced glycation end products (AGEs) and other carbonyl compounds in body tissues (26, 28, 29). The prevalence of atopic diseases has reached epidemic proportions in the 21^st^ century, which is why the role of oxidative, nitrosative and carbonyl stress in selected atopic diseases should be carefully scrutinized. In view of the general scarcity of studies summarizing the latest knowledge in this area, the aim of this article was to review the literature on disruptions in redox homeostasis in allergy patients.

## Respiratory allergies

### Allergic asthma and oxidative stress

**Figure 1:**
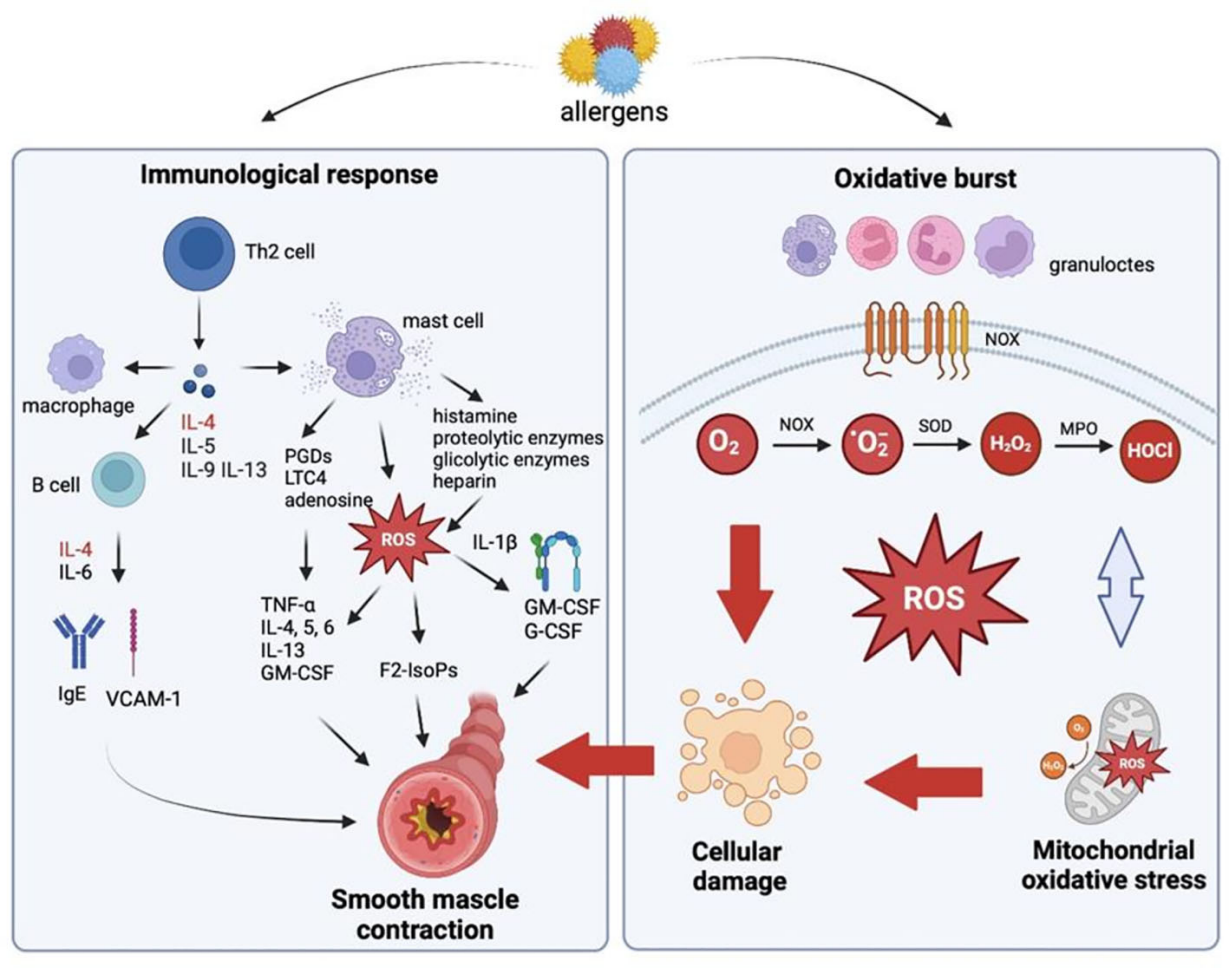
Oxidative stress-mediated inflammation in allergic diseases (Created in Biorender.com). Activated Th2 cells produce interleukin (IL)-4 (IL-4), -5 (IL-5), -9 (IL-9), -13 (IL-13), and the granulocyte-macrophage colony-stimulating factor (GM-CSF) which affect the target cells of allergic reactions such as B cells, mast cells, eosinophils, and macrophages. Histamine, proteolytic enzymes, glycolytic enzymes, and heparin, as well as *de novo* synthesized prostaglandin D2 (PGD2), leukotriene C4 (LTC4), adenosine, and reactive oxygen species (ROS) are released from mast cell granules, and they directly induce oxidative stress. These mediators cause airway smooth muscle contraction, sensitize nerve terminals, promote mucus hypersecretion, vasodilation, and plasma extravasation from microvessels. In allergy, spontaneous or stimulated overproduction of ROS in mast cells, eosinophils, neutrophils, macrophages and monocytes is also observed. The endothelial enzyme NADPH oxidase (NOX) activated by airborne allergens are a rich source of superoxide anions (O_2_^•-^). O_2_^•-^ radicals are not highly reactive or toxic, but they are rapidly converted to hydrogen peroxide (H_2_O_2_). H_2_O_2_ is used mainly by myeloperoxidase (MPO) which catalyzes oxidation reactions with the involvement of chlorine ions to produce hypochlorous acid (HOCl), hydroxyl radicals (OH^•^), singlet oxygen (O_2_^1^), and ozone (O_3_) with strong bactericidal properties. ROS increase the severity of allergic diseases by activating numerous inflammatory mediators which cause bronchial hyperreactivity by contracting the airway smooth muscle, promoting mucus hypersecretion, increasing the permeability of pulmonary capillaries, and damaging cell membranes. B cell, lymphocyte type B; F2-IsoPs- F2-isoprostanes; G-CSF, granulocyte colony-stimulating factor; GM-CSF, granulocyte-macrophage colony-stimulating factor; H_2_O_2_, hydrogen peroxide; HOCl, hypochlorous acid; IgE, immunoglobulin E; IL, interleukin; LTC4- leukotriene C4; MPO, myeloperoxidase; NOX, NADPH oxidase; O_2_, oxygen; O2•, superoxide anion; PGDs, prostaglandins; ROS, reactive oxygen species; SOD, superoxide dismutase; Th cell, T helper cell; TNF-α, tumor necrosis factor α; VCAM-1, vascular cell adhesion molecule 1.

Bronchial asthma is an inflammatory disorder of the lower respiratory tract which usually occurs in childhood and persists throughout life. According to epidemiological research, bronchial asthma is caused mainly by hypersensitivity to inhalant allergens (3). Allergy-induced asthma is observed in around 70-90% of asthma patients. In all types of asthma, the immune response involves mainly T cells, but according to some reports, regulatory T cells are also implicated in the progression of asthma and other allergic diseases (30). Activated Th2 cells produce IL-4, IL-3, IL-5, IL-13, and the granulocyte-macrophage colony-stimulating factor (GM-CSF) which affect the target cells of allergic reactions such as B cells, mast cells, eosinophils, and macrophages (**Figure 1**). These cells secrete various mediators that are directly responsible for the progression of allergy and bronchial obturation in asthma (31). Interleukin 4 is produced not only by Th2 cells, and it plays a key role in the initiation of an allergic response. This cytokine causes isotype switching to IgE in B cells, increases the expression of the vascular cell adhesion molecule 1 (VCAM-1), controls Fc-e IgE expression, cytokine and chemokine receptors, and the number of leukocytes that participate in the allergic inflammatory cascade. It should also be noted that IL-4 promotes the differentiation of Th0 cells into Th2 cells, exacerbates the inflammatory response, and leads to IgE production in the absence of an allergen. Interleukin 6 potentiates IgE production by enhancing the effects of IL-4, and increased IL-6 synthesis in asthma patients has been well documented (32, 33). There are two types of asthmatic responses: early and late. The early-phase response occurs 10-20 minutes after allergen inhalation, and it causes bronchoconstriction. Inflammatory mediators such as histamine, proteolytic enzymes, glycolytic enzymes, and heparin, as well as *de novo* synthesized prostaglandin D2 (PGD2), leukotriene C4 (LTC4), adenosine, and ROS are released from mast cell granules (34), and they directly induce oxidative stress (**Figure 1**). These mediators cause airway smooth muscle contraction, sensitize nerve terminals, promote mucus hypersecretion, vasodilation, and plasma extravasation from microvessels. F2-isoprostanes (F2-IsoPs) are potent vasoconstrictors (35)These compounds are similar to prostaglandins, and they are produced during non-enzymatic ROS peroxidation of polyunsaturated fatty acids (PUFAs), mainly arachidonic acid. F2-IsoPs are directly responsible for airway smooth muscle contraction and plasma extravasation from the vascular bed (36). Under exposure to IL-1β, F2-IsoPs release GM-CSF and the granulocyte colony stimulating factor (G-CSF) (37). F2-IsoPs also activate the PGF2α receptor, which inhibits air flow due to bronchial obturation and leads to respiratory distress (36). At the same time, mast cells secrete cytokines that directly trigger the late-phase asthmatic response (TNF-a, IL-4, -5, -6) and recruit other inflammatory cells (IL-13 and GM-CSF). Bronchial contractions that occur 6-12 hours later are known as the late-phase asthmatic response. According to research, the late phase has a much greater impact on the pathogenesis of asthma than the early phase (36). The mediators secreted by mast cells trigger the expression of adhesion molecules in the endothelium of vessels responsible for leukocyte migration to the inflammation site (31). ROS intensify T cell activation and induce leukocyte adhesion to the endothelium, thus recruiting circulating leukocytes to the inflammation site (11). The following adhesion molecules play a key role in cell transmigration and activation in asthma: intercellular adhesion molecule 1 (ICAM-1), VCAM-1, and its ligand VLA-4. Adhesion molecules bind to their ligands present on leukocytes, which enables them to closely adhere to the microvascular endothelium. In a process known as diapedesis, cells respond to chemokines and cross the endothelial barrier to reach the inflammation site (38). The number of eosinophils, neutrophils, and CD4 and CD8 T cells increases in the late-phase asthmatic response. Mast cells also migrate to the epithelium. Recent research has shown that epithelial cells play a role in inflammation (39). Cytokines produced by Th2 cells secrete chemokines that induce and sustain inflammation in tissues (40). There is also evidence to suggest that epithelial cells promote the differentiation of Th2 cells (41, 42). Prolonged inflammation leads to bronchial hyperreactivity to non-specific stimuli. Migrating inflammatory cells are activated by the released or locally produced factors, and they become hypersensitive to various stimuli. The activation of inflammatory cells leads to the release of other mediators that cause smooth muscle contraction, hyperemia, edema and damage to respiratory mucosa, and stimulation of nerve terminals (43).

There is considerable evidence that oxidative stress increases the severity of bronchial asthma (43–45). Spontaneous or stimulated overproduction of ROS in mast cells, eosinophils (46), neutrophils (47), macrophages (48) and monocytes (38) is observed in asthma. Activated phagocytes utilize ROS to eliminate allergens. This process is known as respiratory burst, and it increases oxygen consumption in cells several dozen-fold. The endothelial enzyme NOX plays a key role in respiratory burst (49). NOX is activated by airborne allergens that are a rich source of this enzyme (36). Overexpression of NOX generates superoxide anion radicals (O_2_^•-^). O_2_^•-^ radicals are produced when electrons from NADPH produced in the pentose phosphate pathway are converted to molecular oxygen (O_2_) in extracellular fluid or inside phagosomes. O_2_^•-^ radicals are not highly reactive or toxic, but they are rapidly converted to hydrogen peroxide (H_2_O_2_). This compound is more stable than O_2_^•-^, and it can penetrate cell membranes (50). Hydrogen peroxide produced by neutrophils is used mainly by MPO which catalyzes oxidation reactions with the involvement of chlorine ions to produce hypochlorous acid (HOCl), hydroxyl radicals (OH^•^), singlet oxygen (O_2_^1^), and ozone (O_3_) with strong bactericidal properties (49). ROS are also produced in the Haber-Weiss reaction (O_2_^•-^+ H_2_O_2_ → OH^•-^ + OH^-^ + O_2_) and the Fenton reaction (H_2_O_2_ + Fe^2+^ → Fe^3+^+ OH^•-^ + OH^-^) with the participation of pro-oxidant ions of transition metals (mostly Fe^2+^/Cu^2+^) (51). Inflammatory cells produce much larger quantities of ROS/RNS under allergic conditions than in healthy subjects (52). Acidophilic granulocytes are immune cells with the greatest potential for ROS generation (46).

In bronchial asthma, ROS act as mediators of asthmatic inflammation and deliver direct pro-asthmatic effects (53, 54). ROS increase the severity of bronchial asthma by activating numerous inflammatory mediators, including leukotrienes B4 (55) and D4, regulated on activation, normal T-cell expressed and secreted (RANTES) chemokines, eotaxins, IL-5 (55), IL-1, -4, -6, GM-CSF, interferon (INF), platelet-activating factor (PAF), tumor necrosis factor α (TNFa), adhesion molecules (VCAM-1) (56) and major basic protein (MBP) (57). Inflammatory mediators cause bronchial hyperreactivity by contracting the airway smooth muscle, promoting mucus hypersecretion, increasing the permeability of pulmonary capillaries, and damaging cell membranes (58). Pro-inflammatory mediators are overexpressed via the mitogen-activated protein kinase (MAPK), nuclear factor kappa B (NF-KB), and activator protein 1 (AP-1) signaling pathways which not only regulate the inflammatory response, but also intensify ROS production and oxidative stress (59–61). These processes further disrupt redox homeostasis (62). Oxidative stress causes cell membrane oxidation in the lipid peroxidation process, which alters the physical properties of cell membranes, induces changes in membrane fluidity and cell integrity. The activity of membrane enzymes and transport proteins is also inhibited (7). Protein oxidation leads to the cleavage of the polypeptide chain, changes in amino acid residues, and the formation of dimers and/or protein aggregates, which disrupts the functions of structural and transport proteins. Oxidative stress also causes breaks in the DNA strand, which leads to changes in gene expression and genetic mutations (8, 63). Indeed, ragweed pollen extract depleted cellular GSH and induced lipid peroxidation, which directly induced tyrosine phosphorylation of p38 MAPK and subsequent production of interleukin IL-8. The resulting oxidative stress induced mucous cell metaplasia and the accumulation of inflammatory cells in bronchoalveolar lavage (BAL) fluids and peribronchial areas (64). Pollen glucans can also stimulate Toll-like receptors (TLRs) in phagocytes and dendritic cells (DCs), and they can contribute to mitochondrial dysfunctions that also lead to the overproduction of ROS (65). Ragweed pollen extract caused oxidative damage to ubiquinol-cytochrome c reductase core protein II (UQCRC2) and intensified the release of hydrogen peroxide from mitochondrial complex III (66). Mitochondria are an important source of ROS in most cells. Therefore, increased production of mitochondrial ROS can modify allergic airway inflammation and bronchial hyperreactivity (65, 66). It was shown that mitochondrial oxidative stress occurs mainly during hypoxic conditions. Hypoxia-inducible factor 1α (HIF-1α) is a crucial transcription factor modulating the high energy requirements of immune cells to perform their effector functions in low-oxygen states (67–70). Hypoxia increases mitochondrial ROS formation and leads to enhanced infiltration of activated inflammatory cells (71). A better understanding of the role of hypoxia in immunological reactions may result in developing new therapeutic strategies for allergic diseases (67).

The toxic effects of ROS are mitigated by antioxidant enzymes, including catalase (CAT), glutathione peroxidase (GPx), superoxide dismutase (SOD), as well as non-enzymatic antioxidants such as glutathione (GSH), vitamins C and E, and uric acid. In asthma, oxidative stress can be caused by the weakening of enzymatic and non-enzymatic antioxidant mechanisms that accompany chronic inflammations (72) (**Table 1**). In asthma patients, lower levels of SOD, CAT (73) and GPx (74) activity were observed in BAL fluids and respiratory epithelial cells. A significant decrease was also noted in the plasma concentrations of non-enzymatic antioxidants: hydrophilic ascorbic acid and hydrophobic α-tocopherol. The activity of salivary antioxidant enzymes (salivary peroxidase, SPO; SOD) was also significantly lower in asthma patients (75). A marked decrease was also reported in the serum levels of antioxidant vitamins (C and E) (76–78). A weakened antioxidant barrier contributes to oxidative changes in cellular biomolecules. Orally administered antioxidants (vitamins C and E) decreased bronchial hyperreactivity in asthma patients (79) and reduced the consumption of inhalation corticosteroids (80). In an experimental study, antioxidants decreased the accumulation of acidophilic granulocytes in the respiratory tract of animals with inhalant allergies (81). For this reason, antioxidant supplements are recommended in the treatment of asthma. The efficacy of antioxidant enzymes in asthma treatment has been also investigated, and a study of liposome-encapsulated SOD and CAT generated promising results (82). Liposome-encapsulated enzymes decreased bronchial hyperreactivity in the experimental animals. Attempts have also been made to synthesize SOD mimetics, and initial results suggest that these enzymes have therapeutic potential in asthma (83).

**Table 1:** Redox biomarkers in patients with allergic diseases.

Study group	Control group	Marker	Material	Changes vs control group	Reference	Comments
Asthma						
15 asthmatic patients (M/F-8/7) <18 years – 9 ≥18 years - 6	15 healthy subjects (M/F-8/7) <18 years – 9 ≥18 years - 6	8-iso-PGF2alpha (EIA)	Plasma	Increase (p 0.0044)	(2000) Wood LG (85).	8-iso-PGF2α levels were found to be associated with clinical asthma severity (P = 0.044) (R=0.367)
12 asthmatic children undergoing steroid treatment (M/F-5/7) (5-17 years)	11 healthy children (M/F-8/7) (2-18 years)	8-iso-PGF2alpha (CL)	Exhaled breath condensate	Increase (p < 0.01)	(2005) Shahid SK (86).	Inhaled corticosteroids were administered at ≤ 600 μg/day to 6 children and at >600 μg/day to 6 children
12 asthmatic children undergoing non-steroid treatment (M/F-4/8) (5-17 years)	11 healthy children (M/F-8/7) (2-18 years)	8-iso-PGF2alpha (CL)	Exhaled breath condensate	Increase (p < 0.01)	(2005) Shahid SK (86).	10 patients with mild intermittent asthma and 2 patients with moderate persistent asthma
30 asthmatic children (M/F-19/11) (4-15 years)	21 healthy children (M/F-14/15) (5-13 years)	carbon monoxide (chemical analyzer)	Exhaled breath condensate	increase (p<0.01)	(2002) Zanconato S (112).	Nineteen (63%) of the children had persistent asthma and were undergoing a long-term treatment with low doses of inhaled steroids for at least 1 month; 7 children (23%) were also administered long-acting β2 agonists. The remaining 11 children had intermittent asthma and did not receive long-term treatment
29 asthmatic children (M/F-18/11) (4-15 years)	23 healthy children (M/F-11/12) (5-13 years)	nitric oxide (CL)	Exhaled breath condensate	increase (p<0.0001)	(2002) Zanconato S (112).	Children were treated with oral prednisone for 5 days (1 mg/kg/day)
50 patients with asthma (M/F -25/25) (15- 65 year)	healthy subjects (M/F -25/25) (15- 65 year)	MDA (spectrophotometry)	Plasma	increase (p ≤ 0.05)	(2014) Abeer M.A (271)	
12 children during an asthma episode (6-18 years)	14 healthy children (6-16 years)	SPO, TSA, SOD (NBS)	Saliva	decrease (p<0.05)	(2006) Bentur L (75).	No correlation was found between saliva antioxidant levels
15 asthmatic patients	16 healthy subjects	total carotenoids, lycopene, lutein, β-cryptoxanthin, β-carotene, and α-carotene (HPLC)	Sputum, whole blood	decrease (p<0.001)	(2005) Wood LG (76).	
81 asthmatic patients (M/F -27/54) (44–58 years)	43 healthy subjects (M/F -19/23) (36-56 years)	Vitamin C, α-tocopherol, retinol, β-carotene, α-carotene, lycopene (HPLC)	Plasma	decrease (p<0.001)	(2005) Misso LA (77).	53 patients with mild to moderate asthma and 28 patients with severe asthma
8 patients with mild asthma	6 healthy subjects	NF-κB (ELISA)	BAL	decrease (p<0.05)	(1999) Thomassen MJ. (98)	
24 patients with mild asthma (M/F -18/6) (21–33 years)	24 healthy subjects (M/F -13/11) (22–32 years)	NO (WB)	Exhaled breath condensate	increase (p<0.001)	(2003) Hansel TT (106).	All study and control group patients received 200 mg of SC-51 before the study
38 asthmatic patients (M/F -21/17) (22–63 years)	32 subjects (M/F -18/14) (20–63 years)	leukocyte GPx, vitamin C (spectrophotometry)	Serum	decrease (p<0.01)	(2005) Vural H (78).	All patients had mild asthma without acute episodes in the past 3 months. They regularly took inhaled corticosteroids at low doses and short-acting β2 agonist when needed.
81 asthmatic patients (M/F -27/54) (44–58 years)	43 healthy subjects (M/F -19/23) (36-56 years)	Vitamin C, α-tocopherol, retinol, β-carotene, α-carotene, lycopene (HPLC)	Plasma	decrease (p<0.001)	(2005) Misso LA (77).	53 patients with mild to moderate asthma and 28 patients with severe asthma
Allergic rhinitis						
40 patients (M/F -18/22) (10–53 years)	40 patients (M/F -16/24) (13–48 years)	AOPPs	Serum	increase (p=0.0015)	(2009) Aksoy F (133).	Patients with allergic rhinitis received subcutaneous immunotherapy (SCIT)
100 children with asthma and allergic rhinitis (M/F -37/63) (8-13 years)	74 healthy children (M/F -42/32) (8-12 years)	MDA (HPLC)	Exhaled breath condensate (nasal and oral)	increase (p < 0.01)	(2012) Celik M (136).	Nasal and oral MDA (r = 0.684, p < 0.001) were positively correlated with GSH concentrations (r = 0.684, p < 0.001). Negative correlations were found between GSH and MDA levels in both nasal (r = 0.573, p < 0.001) and oral samples (r = 0.387, p < 0.001)
17 children with allergic rhinitis (M/F -9/8) (10-14 years)	74 healthy children (M/F -42/32) (8-12 years)	MDA (HPLC)	Exhaled breath condensate (nasal and oral)	increase (p < 0.01)	(2012) Celik M (136).	
100 children with asthma and allergic rhinitis (M/F -37/63) (8-13years)	74 healthy children (M/F -42/32) (8-12 years)	GSH (HPLC)	Exhaled breath condensate (nasal and oral)	decrease (p < 0.01)	(2012) Celik M (136).	
17 children with allergic rhinitis (M/F -9/8) (10-14 years)	74 healthy children (M/F -42/32) (8-12 years)	GSH (HPLC)	Exhaled breath condensate (nasal and oral)	decrease (p < 0.01)	(2012) Celik M (136).	
70 patients with allergic rhinitis (mild and moderate)	24 healthy subjects	MDA (spectrophotometry)	Exhaled breath condensate	increase (p < 0.01)	(2021) Gadzhimirzaev GA (139).	
11 patients with allergic rhinitis (M/F -8/3) (15-56 years)	18 patients with non-allergic rhinitis (M/F -17/1) (15-56 years)	Nitrotyrosine (immunohisto- chemistry)	nasal mucosa	increase (p<0.05)	(2000) Kang BH (135).	Allergic rhinitis patients who required turbinectomy
93 patients with severe allergic rhinitis (20-22 years)	40 non-allergic subjects (24-48 years)	Nitrotyrosine (HPLC)	nasal mucosa	increase (p<0.05)	(1998) Sato M (134).	
6 patients with allergic rhinitis (M/F -1/5) (26-54 years)	13 non-allergic subjects	Carbon monoxide (infrared analyzer)	nasal airways	increase (p<0.005)	(2002) Andersson JA. (138)	
86 patients with allergic rhinitis (M/F -32/54) (37-41 years)	30 non-allergic subjects (M/F -34/40) (26-54 years)	carbon monoxide (EC50 analyzer)	Exhaled breath condensate	increase (p<0.005)	(1999) Monma M (137).	
Atopic dermatitis						
21 patients with atopic dermatitis (M/F -14/7) (21-76 years)	20 healthy subjects (M/F -16/4) (25-95 years)	Nitrate (Griess method)	urine	increase (p<0.05)	(2009) Nakai K (25).	MDA and 8-OHdG were also measured. Urinary nitrate levels, but not 8-OHdG or malondialdehyde, were significantly higher in atopic dermatitis patients than in healthy controls. The Eczema Area and Severity Index (EASI) score was significantly correlated with urinary nitrate (r = 0.647, P = 0.03) and urinary malondialdehyde levels (r = 0.472, P = 0.03). The EASI score was not correlated with urinary 8-OHdG levels
17 patients (M/F -4/13) (16-36 years)	17 healthy subjects (M/F -4/13) (19-34 years)	8-OHdG (ELISA)	Urine	increase (p< 0.0001)	(1998) Tsuboi H (152).	
13 children with AD (M/F -4/9) (8.5-11.5 years)	28 healthy children (M/F -10/18) (1.5-10 years)	8-OHdG, acrolein-lysine adducts, BOM (bilirubin oxidative metabolites) (ELISA)	Urine	increase (p<0.001)	(2003) Tsukahara H (150).	
33 children with AD (2.5-6 years)	23 healthy children (2.5-5.5 years)	8-isoprostane, LTB4 (EIA)	Exhaled breath condensate	increase (p<0.01)	(2009) Peroni DG (272).	
15 patients with AD (M/F -7/8) (27-33 years)	40 healthy subjects (M/F -20/20) (29-35 years)	carbon monoxide (electrochemical analyzer)	Exhaled breath condensate	increase (p<0.0001)	(1999) Horvath I (151).	
75 patients with AD (M/F -37/38) (20-53 years)	15 healthy controls and 22 diseased controls, including 12 patients with contact dermatitis and 10 patients with cutaneous dermatophyte infection or cutaneous candidiasis	4-hydroxy-2-nonenal, carbonyl moieties, SOD (spectrophotometry)	skin biopsies	increase (p<0.05)	(2003) Niwa Y (155).	
75 patients with AD (M/F -37/38) (20-53 years)	15 healthy controls and 22 diseased controls, including 12 patients with contact dermatitis and 10 patients with cutaneous dermatophyte infection or cutaneous candidiasis	Vitamin E (MS)	skin biopsies	decrease (p<0.05)	(2003) Niwa Y (155).	
5 patients with AD (23-46 years)	5 healthy subjects	Vitamin C (MS)	skin biopsies	decrease (p<0.05)	(2003) Leveque N (154).	
Urticaria						
16 patients with urticaria (20-63 years)	11 healthy subjects	MDA, SOD, GSH (immuno-dot blot assay)	skin biopsies	increase (p<0.05)	(2003) Raho G (164).	
85 patients with urticaria (M/F -31/54) (16-77 years)	64 healthy subjects	AOPPs (spectrophotometry)	Serum	increase (p<0.0001)	(2017) Nettis E (167).	AGEs were also measured. No significant differences were found between AGE levels in patients and controls.
31 children with urticaria (M/F -16/15) (7-15 years)	22 healthy children (M/F -10/12) (7-13 years)	NOx metabolites (nitrite, nitrate) (Greiss method)	Serum	increase (p<0.0001)	(2017) Dilek F (27).	
25 patients with urticaria (M/F -6/19) (mean age 35 years)	20 healthy subjects	Vitamin E (MS), catalase, CSH-Px (spectrophotometry)	Plasma	decrease (p<0.0001)	(2001) Briganti S (148).	

8-iso-PGF2alpha, 8-iso-Prostaglandin; 8-OHdG, 8-hydroxy-2’-deoxyguanosine; AD, atopic dermatitis; AOPPs, advanced oxidation protein products; BOM, bilirubin oxidative metabolites; CL, chemiluminescence; GPx, glutathione peroxidase; EISA, Eczema Area and Severity Index; EIA, enzyme immunoassay; ELISA, enzyme-linked immunosorbent assay; GSH, glutathione; HPLC, high-performance liquid chromatography; LTB4, leukotriene B4; MDA, malondialdehyde, MS, mass spectrometry; NBS, thionitrobenzoic acid assay; NOx, nitrogen oxides; SOD, superoxide dismutase; SPO, salivary peroxidase; TSA, thiol-specific antioxidant protein; WB, Western Blot.

Oxidative stress plays a key role in the pathogenesis of asthma, which is why redox biomarkers could have considerable diagnostic potential in asthma (**Table 1**). ROS production rate was also correlated with disease severity (84). Inhaled corticosteroids were found to decrease ROS concentrations in the respiratory tract. Peripheral eosinophil counts were significantly higher in asthma patients than in the control group, and were correlated with disease severity. F2-IsoPs deserve special attention because serum F2-IsoPs levels were higher in asthma patients than in healthy subjects, and were also correlated with disease progression (85). Other researchers also reported an increase in F2-IsoPs concentrations in the blood serum (78) and exhaled breath condensate of asthma patients (86). F2-IsoPs are stable products of arachidonic acid oxidation, and their levels can increase even six-fold during an acute asthma episode, and decrease in response to corticosteroid therapy (85). F2-IsoPs are effective vasoconstrictors that induce mitogenesis in vascular smooth muscle cells. They also modulate platelet functions (85). In asthma patients, oxidative stress is also manifested by a rise in the levels of malondialdehyde (MDA), a product of lipid peroxidation with cytotoxic and mutagenic properties (87, 88). Elevated MDA levels were reported in the serum, plasma, hemolysate, and exhaled breath condensate of asthma patients relative to healthy controls. Interestingly, MDA concentration increases significantly in acute asthma, which suggests that peroxidation processes are intensified during disease progression (89). Lipid peroxidation in cell membranes stimulates phospholipase enzymes (including phospholipase A2), which triggers the arachidonic acid cascade catalyzed by cyclooxygenases and lipoxygenases (38). ROS damage membrane enzyme complexes and increase the flow of calcium to cells, which contributes to the secondary activation of cellular proteases and phospholipases. These processes damage cell membranes and membrane receptors in the respiratory tract. Despite extensive research, there is considerable debate on whether heightened oxidative stress in the respiratory tract of asthma patients is the cause or consequence of inflammation, oxidative stress preceded asthma symptoms (allergic inflammation and respiratory sensitization) (90). In atopic patients, oxidative stress can initiate intercellular signaling pathways, leading to a break in immune tolerance (61).

### Allergic asthma and nitrosative stress

Nitrosative stress is closely associated with oxidative stress in the progression of asthma. Nitrosative stress leads to the overproduction of RNS, including nitric oxide (NO) which triggers an immune response, and its derivatives: nitrous acid (HNO_2_), nitrogen dioxide (NO_2_), and nitric acid (HNO_3_) (91). NO is biosynthesized with the involvement of NOS enzymes that catalyze the oxidation of the guanidinium group in L-arginine to NO and L-citrulline. There are three isoforms of NOS, where inducible NOS (iNOS, type II NOS) plays the key role in the pathogenesis of asthma (92). This enzyme is expressed on bronchial epithelial cells, endothelial cells, fibroblasts, type II pneumocytes, and infiltrating inflammatory cells (93). In asthma patients, iNOS expression in respiratory epithelial cells and NO concentration in exhaled breath condensate were markedly higher due to ongoing inflammatory processes (94). The expression of iNOS is induced by exposure to allergens, pro-inflammatory cytokines (IL-1β, IFN-γ, TNF-α), bacterial lipopolysaccharide, and pro-oxidants (92). The concentration of NO produced under such conditions can be even 1000 times higher than the concentration of NO produced by constitutive NOS (neuronal NOS, nNOS, or endothelial NOS, eNOS). It should also be noted that significant quantities of O_2_^•-^ are produced in the reactions catalyzed by iNOS, and O_2_^•-^ reacts with NO to generate peroxynitrite (ONOO^–^) with strong oxidizing and nitriding properties (95). The adverse effects of NO on the respiratory tract can be attributed mostly to the production of ONOO^–^ and other RNS (NO_2_) (93).

It should also be noted that NO plays a dual role in the pathogenesis of asthma. NO can act as a vasodilator, neurotransmitter, and immune response regulator (96). However, NO can also induce inflammation by generating toxic RNS (97). According to Tomassen and Raychaudhuri (98), NO plays a protective role in asthma by interacting with NF-KB and modulating the inflammatory response. Research has demonstrated that NO donors inhibit the production of inflammatory cytokines (TNF-α, IL-1, MIP-1α) and NF-KB activation in lung macrophages. The expression of NF-KB in BAL fluids is much lower in asthma patients than in healthy subjects due to greater bioavailability of NO in the respiratory tract (99). The results of an experiment conducted on murine Th1 and Th2 cells suggest that NO can exacerbate the inflammatory response in asthma by disrupting the balance between T helper cells (100). After reacting with the allergen and degranulating mast cells in the early phase, NO downregulates the mediators of smooth muscle contraction and induces smooth muscle relaxation by interacting with cyclic guanosine monophosphate (cGMP) (96). However, NO also increases vascular permeability and promotes mucus secretion from the respiratory tract. This vasodilator increases the availability of inflammatory mediators (TNF-α, L-selectin) which, at high concentrations, can damage epithelial cells and lead to bronchial contraction (101). Research has also shown that NO exerts cytotoxic effects on respiratory epithelial cells and disrupts oxygen uptake by type II pneumocytes (93). NO can also activate matrix metalloproteinases (MMPs) which play a role in lung damage (102). There is a growing body of evidence to suggest that NO participates in airway remodeling in asthma (58, 103). An *in situ* analysis of respiratory epithelial cells from asthma patients revealed that iNOS expression is inhibited by glucocorticoids. The levels of NO in exhaled breath condensate returned to normal after treatment (98). An *in vitro* study revealed that glucocorticoids decrease iNOS expression by inhibiting gene transcription and promoting the degradation of enzyme proteins. Selective iNOS inhibitors are used to remove excess NO from the respiratory tract (104). These compounds can decrease the rate of NO production to constitutive NO production levels (105, 106). Zhang et al. (107, 108) demonstrated that rapamycin and budesonide inhibit iNOS and NO synthesis in asthma patients.

The levels of NO in exhaled breath condensate can have a significant diagnostic value in asthma patients (**Table 1**). Numerous studies have shown that NO is a sensitive biomarker of eosinophil-mediated inflammation and that it is positively correlated with other diagnostic markers in bioptates, BAL fluids, and induced sputum (104, 109, 110). NO is particularly useful for monitoring the effectiveness of inhaled steroids in asthma (111). Exhaled NO measurement is a simple, non-invasive, and safe test that reliably assesses inflammation severity in the lower respiratory tract. Interestingly, increased levels of exhaled NO can be associated with a higher risk of asthma (106), and can point to the coexistence of asthma in patients with chronic obstructive pulmonary disease (COPD) (112).

### Allergic rhinitis and oxidative stress

Allergic rhinitis is yet another atopic disease that is caused by ROS and RNS overproduction. It is an IgE-dependent inflammation of the nasal mucosa under exposure to an allergen. This disease is highly prevalent, and it is a common comorbidity of asthma. Inflammation of the nasal mucosa is caused by complex immune responses to an allergen (113). Under exposure to an allergen, Th2 cells begin to secrete cytokines (IL-4, IL-3) which recruit IgE antibody-producing B cells and cause eosinophils to migrate to the subepithelial layer. Inflammatory mediators are released within seconds due to the overproduction of IgE which binds to mast cells and allergens. Histamine is the key inflammatory mediator, but other substances are also released by mast cells, including PAF, eicosanoids, prostaglandins, leukotrienes, substance P, and ROS. ROS play a particularly important role in inflammation (114). Overproduction of ROS leads to the translocation of NF-κB to the cell nucleus and provides cytokine, chemokine, and growth factor genes with access to promoter regions. Intracellular concentration of Ca^2+^ increases, which triggers an inflammatory response. Similarly to asthma, mast cells, Th2 cells, basophils, and epithelial cells synthesize and release cytokines and chemokines (IL-3, IL-4, IL-5, IL-10, IL-13, GM-CSF, and RANTES), which are responsible for sustaining allergic inflammation and the associated oxidative/nitrosative stress (115). Eosinophils, which are responsible for the late-phase reaction, are mobilized in the next stage. Eosinophils damage epithelial cells, the basement membrane, and nerve terminals via toxic MBP and eosinophil cationic proteins (ECP), which leads to edema of the nasal mucosa (116–118).

Interestingly, dust and gaseous air pollutants can contribute to oxidative and nitrosative stress in AR patients. Airborne substances not only exacerbate, but can also induce inflammations of the upper airway mucosa. Their toxicity is determined by chemical composition, duration of exposure, and the accumulation of these substances in the body. Research has demonstrated that household dust stimulates eosinophils to overproduce hydrogen peroxide (119). Ozone also sensitizes airway mucosa to allergens. Ozone facilitates allergen penetration and intensifies disease symptoms (120–122). In turn, NO_2_ exerts negative effects by increasing the prevalence of both AR and bronchial asthma. This relationship was reported by Italian researchers who observed that increased NO_2_ concentration in combination with high temperature increases the prevalence of allergies (123). Diesel exhaust particles (DEP) also exert harmful effects and significantly contribute to risk of inhalant allergies in children and adults by exacerbating oxidative stress and the resulting inflammations (124). The adjuvant effect of DEP on IgE synthesis induces hypersensitivity to inhalant allergens (125). There is evidence to suggest that DEP increase Th2-mediated immune responses and promote allergic reactions (126). Allergic responses to cigarette smoke may be triggered by the same mechanism. Inhaled elemental carbon ultrafine particles activate the NF-KB signaling pathway, which augments allergen-induced lung inflammation and airway hyperresponsiveness (127). Cigarette smoke also contributes to mitochondrial DNA mutations, which increases ROS production and exacerbates neutrophilic airway inflammation (128). Diesel exhaust particles (DEPs) can also adsorb airborne allergens released by pollen, thus intensifying IgE-mediated immune responses (129). Porębski et al. (130) observed that the prevalence of inhalant allergies was higher in children residing in the immediate vicinity of major road networks.

Redox homeostasis in AR patients has been investigated by very few studies to date (**Table 1**). However, it is believed that oxidative and nitrosative stress in AR is associated with chronic upper and lower airway inflammation (131, 132). Aksoy et al. reported higher concentrations of protein oxidation products in AR patients (133), whereas Hang et al. and Sato (134, 135) observed an increase in 3-nitrotyrosine levels (a product of tyrosine nitration) in severe allergic reactions. Other studies revealed that lipid peroxidation products, mainly MDA, are effective biomarkers of inflammation in the progression of AR (136). Carbon monoxide (CO) levels in exhaled breath condensate were also higher in AR patients (137, 138). Most importantly, these findings indicate that protein and lipid oxidation products can be used as potential diagnostic markers in AR (139).

## Skin allergies

### Atopic dermatitis and oxidative stress

Atopic dermatitis is a chronic recurrent skin disease associated with elevated IgE levels, sensitization to common food and inhalant allergens, and skin barrier defects (3). Patients with AD are strongly predisposed to IgE-mediated sensitivity to exogenous and endogenous antigens in both type I and IV hypersensitivity reactions (140). In this disease, CD4 T cells differentiate mainly into Th2 cells, whereas Th1 proliferation is inhibited (141). In AD patients, the imbalance in T cell populations weakens the local immune response to bacterial and viral infections, increases skin colonization by pathogenic microorganisms, and decreases late-phase hypersensitivity (142). Increased Th2 activity enhances cytokine production, in particular IL-4, IL-5, and IL-13, and decreases IFN- γ synthesis (143). In addition to elevated Th2 levels in peripheral blood and skin, immune cells were also activated by an increase in the number of specific surface receptors (142). In AD, cytokines overproduce IgE and contribute to allergic inflammation (3). The production of ROS and RNS also increases, which enhances cytokine and chemokine secretion (140). IgE-mediated antigen presentation plays an important role in the pathogenesis of atopy despite the fact that serum IgE levels in AD patients are frequently uncorrelated with the severity of disease symptoms (144). IgE receptors are present in many immune cells, and their expression is higher in AD patients than in healthy subjects. These receptors increase the antigen-presenting capacity of Langerhans cells hundreds or even thousands of times. IgE-specific receptors are also found in T and B cells, monocytes, macrophages, eosinophils, and blood platelets (145). For this reason, IgE-mediated reactions are triggered by various mechanisms (140). Elevated IgE antibody levels were noted in around of 80% AD patients, and IgE specific for common inhalant and food allergens were also detected in most patients (144). Inhalant and food allergens easily penetrate mucous membranes, enter the blood stream, and cause skin lesions (146). Skin barrier dysfunction is observed in AD patients, and allergens can easily reach deeper skin layers to stimulate sensitized memory cells via antigen-presenting cells (APCs) (3, 147). Circulating IgE autoantibodies against self-proteins have been identified in most AD patients. The presence of IgE autoantibodies can partly explain the chronic and recurrent character of AD, as well as problems with identifying allergens that cause skin lesions (144). A polyclonal response to superantigen-producing bacteria and fungi that often colonize the skin has been also observed in AD patients. These superantigens do not have to be stimulated by Langerhans cells, and they can activate many lymphocytes via non-specific receptors (3).

Briganti et al. (148) reported dense lymphocytic, monocytic, and eosinophilic infiltrates in skin bioptates of AD patients. These cells generate large amounts of O_2_^•-^ and NO• which damage skin cells. The skin acts as a physical barrier between the internal and the external environment. It protects the body against UV radiation, mechanical damage, xenobiotics, and microorganisms, and it is highly susceptible to oxidative damage (149). The concentrations of lipid peroxidation products (acrolein-lysine adducts) are thus higher in AD patients (150). Isoprostanes are also robust indicators of oxidative stress. Increased 8-isoprostane levels in various biological materials (plasma, BAL fluids, exhaled breath condensate, urine) are indicative of intensified lipid peroxidation in AD patients (150). Exhaled CO levels were also found to be higher in AD patients than in healthy controls (151). Overproduction of ROS can also lead to DNA damage. Tsuboi et al. (152) reported an increase in daily urine levels of 8-hydroxy-2’-doxyguanosine (8-OHdG, a marker of DNA damage) in AD patients, which points to oxidative stress. Nucleic acids are least susceptible to oxidative damage because they feature highly effective repair mechanisms and are localized inside cells (153).

In skin allergies, oxidative stress can be intensified by the weakening of endogenous antioxidant defense mechanisms (**Table 1**). Vitamin C and E concentrations are lower in AD patients, which further promotes OH generation and lipid peroxidation (154, 155). Research has shown that N-acetyl-L -cysteine (NAC) and GSH strongly inhibit IL-4 production by Th2 cells and exert a minor inhibitory effect on IL-5 and INF synthesis, which indicates that these compounds are useful in the treatment of Th2-associated diseases, including AD (156–158).

### Atopic dermatitis and nitrosative stress

The role of RNS in the pathogenesis of skin allergies has been examined less extensively than in bronchial asthma (62). Nakai et al. (25) indicate that nitrate concentrations were significantly higher in the urine of AD patients and correlated with disease progression. Indeed, the Eczema Area and Severity Index (EASI) score was significantly correlated with urinary nitrate (r = 0.647, P = 0.03). The researchers request that nitrates may be a valuable biomarker of nitrosative stress in AD patients. In another study (159), Kubo et al. investigated the contribution of NO to the growth of skin lesions in an animal model for AD. The expression of iNOS was increased, while the nNOS expression was decreased in the epidermal skin lesions. Moreover, the immunohistochemical localization of nitrotyrosine was noticed in nearly eosinophils. It is therefore suggested that RNS generation in eosinophils may be connected with AD’s pathogenesis. Further research is needed to clarify the contribution of nitrosative stress to skin allergies.

### Urticaria and oxidative stress

Allergic urticaria is one of the most common skin diseases that affects 15-20% of the global population (3). Urticaria is a heterogeneous group of skin disorders provoked by various exogenous and endogenous factors. The disease is caused by the release of inflammatory mediators from mast cells during an immune response involving specific IgE, as well as food, inhalant, and contact allergens (160). The main inflammatory mediator is histamine, responsible for the sudden outbreak of swollen bumps or plaques on the skin (161), and a similar role is played by PGD2. In turn, the late-phase allergic response is triggered by leukotriene B4 (LTB4), neutrophil chemotactic factor (NCF), eosinophil chemotactic factor of anaphylaxis (ECF-A), and PAF. The late-phase response involves neutrophils and eosinophils, followed by macrophages and lymphocytes. Mast cell serine proteases, tryptase, chymase, and carboxypeptidase, further contribute to mast cell degranulation (161). The Hageman factor and complement and kinin systems are activated, which leads to collagen degradation and increases the permeability of cutaneous vessels (162). These processes contribute to ROS and RNS overproduction and oxidative stress which intensifies cytokine secretion in keratinocytes (163). Keratinocytes produce numerous inflammatory cytokines, including IL-1, IL-3, IL-6, IL-8, GM-CSF, and TNF-α. Keratinocytes interact with immune cells recruited to the site of inflammation and increase the expression of adhesion molecules, which enables immune cells to adhere to the vascular endothelium (3).

According to the literature, oxidative and nitrosative stress plays a role in the progression of chronic spontaneous urticaria (CSU) (**Table 1**) (164, 165). In CSU patients, skin lesion biopsies revealed higher activity of macrophages and basophils which are responsible for ROS and RNS overproduction (166). Oxidative stress markers, including MDA, were significantly elevated and correlated with reduced synthesis of GSH, the most important skin antioxidant (164). Nettis et al. (167) examined advanced oxidation protein products (AOPPs) and AGEs and found that plasma AOPP levels were considerably higher in urticaria patients than in the control group. However, no significant changes in AGE concentration were observed, which suggests that proteins are intensively oxidized, but not glycated in urticaria (166). During oxidative stress, ROS directly interact with amino acid side chains (mainly lysine, arginine, proline, and threonine), or peptide bonds in the polypeptide chain are broken and carbonyl groups are formed inside the polypeptide structure (168). AOPPs are the end products of these processes. ROS also interact with unsaturated fatty acids in phospholipids, and aldehydes such as 4-hydroxynonenal, 4-hydroxyhexanal, acrolein, and MDA are the most reactive compounds (87). These compounds can interact with the amino acid residues of proteins, mainly histidine, cysteine, and lysine, to form carbonyl derivatives known as advanced lipoxidation end-products (ALEs) (169). The accumulation of glucose and its metabolites in cells, for example in diabetes patients, enables this monosaccharide to interact with amino acid residues, which leads to the production of AGEs (168). In urticaria, redox imbalance can be caused by dysfunction of the enzymatic and non-enzymatic antioxidant barrier. A decrease in the levels and activity of antioxidants, including vitamin E, GSH, CAT, and GPx, were indeed noted in urticaria patients (170). However, elevated SOD activity was observed in some patients (171), which points to an adaptive response to ROS overproduction. SOD is the main antioxidant enzyme that eliminates the first product of one-electron reduction of molecular oxygen (O_2_^•-^). In a dismutation reaction, SOD converts O_2_^•-^ to hydrogen peroxide which is then removed by CAT, GPx, and metallothioneins. Therefore, SOD is regarded as the first line of defense against the toxic effects of ROS (172). An increase in SOD activity points to an adaptive response in urticaria patients, but the reserves of other antioxidants that scavenge ROS can become depleted as the diseases progresses. Further research is needed to examine these processes (164).

### Urticaria and nitrosative stress

Dilek et al. (27) examined the possible role of nitrosative stress in urticaria progression. They reported higher plasma levels of NOx metabolites (nitrates and nitrites) in 22 children with chronic spontaneous urticaria. They also found a positive correlation between NOx metabolites and disease progression measured by urticaria activity score (UAS) (27). Further research is needed to clarify the effects of nitrosative stress in urticaria.

## Food allergies

### Pathogenesis of food allergies

A food allergy is an immune response in persons who are highly sensitized to substances that occur naturally in food products (3). These individuals produce IgE antibodies against food ingredients that are well tolerated by the general population (173). IgE-mediated food allergies have numerous symptoms, including systemic reactions (anaphylaxis), acute or chronic skin symptoms (urticaria, AD, angioedema), respiratory symptoms (asthma, rhinitis, laryngitis, ear inflammation), and gastrointestinal symptoms (abdominal pain, nausea, diarrhea, bloating, loss of appetite, vomiting) (174). Neurological symptoms such as headaches and sleep disorders can be also associated with food allergies (175). Symptoms can manifest already several minutes after allergen ingestion, or after several hours or days in late-phase reactions (164).

Gut-associated lymphoid tissue (GALT), which is a component of mucosa-associated lymphoid tissue (MALT), i.e. the mucosal immune system, plays a key role in food allergies (176). Foodborne allergens are transported by M cells from the intestinal lumen to lymphatic tissue beneath the epithelium (177). A food tolerance response or a food allergy (food intolerance) can occur at this stage (3). Allergens transported across the surface of mucous membranes are captured by APCs which engulf and process antigens and present them to T cells. Antigens are bound by class II major histocompatibility complex (MHC) proteins on the surface of APCs (178). Cytokine signaling and the interactions between adhesion molecules and receptors on APCs (ICAM-1, LFA-3) and lymphocytes (LFA-1, CD-2) play a key role in this process. Activated Th0 cells give rise to Th1 and Th2 cell subpopulations (173). Th1 cells can produce interferon- γ (IFN- γ) which inhibits the proliferation and activity of Th2 cells. Th1 cells differentiate from Th0 cells in the presence of IL-12 which is produced manly by mast cells and APCs, i.e. macrophages and DCs (IL-12 is not produced by B cells). The above leads to the overproduction of ROS and RNS, and it intensifies cytokine and chemokine secretion via a positive feedback loop (174). Th1 cells promote the delayed cell-mediated immune response, but they can also stimulate B cells to produce antibodies when their ratio is appropriate. The generation of Th2 cells is stimulated by IL-4 which is secreted by antigen-stimulated Th2 cells, but also by mast cells and basophils (176). Th2 cells produce IL-4, IL-10, and IL-13, which inhibit Th1 differentiation and sustain and enhance the humoral immune response (immediate response). Under the influence of IL-4 and IL-13 and the interactions between adhesion molecules on B cells (class II MHC molecules: CD80/CD86, CD40, and ICAM-1) and T cells (CD3, CD4, CD28, CD40L, and LFA-1 receptors), B cells switch from expressing IgM, IgG2, and IgG3 to IgG4 and IgE (179). This mechanism is responsible for the production of antigen-specific IgE (**Figure 1**) (180). Stimulated B and T cells can migrate to the blood (IgE and the allergen are distributed throughout the body with bodily fluids), and they are transported back to mucous membranes and the associated lymphatic structures (mainly GALT) where the allergen was first encountered. This mechanism is responsible for allergic reactions not only in the gastrointestinal tract, but also in distant organs such as the skin or lungs (173).

An allergen that binds to at least two IgE antibodies reaches a sensitized mast cell and leads to its degranulation via an antigen bridge. Other activators can also participate in mast cell degranulation. For example, strawberries contain lectins that cross-link adjacent IgE molecules and provoke urticaria symptoms in some consumers, in particular children (181). Mast cell degranulation leads to the release and *de novo* synthesis of many mediators, including ROS. These substances can be also produced by basophils, eosinophils, and macrophages under the influence of cytokines secreted by Th2 (IL-3, IL-4, IL-5, IL-13, GM-CSF) (182, 183). The resulting mediators and enzymes, such as leukotrienes, prostaglandins, histamine, tryptase, kininogenase, chymase, and toxic basic proteins (MBP; ECP; eosinophil peroxidase, EPO; eosinophil-derived neurotoxin, EDN), are responsible for allergic reactions, including anaphylaxis (180). Increased EPO activity in food allergies deserves special attention. This enzyme catalyzes the reaction between H_2_O_2_ and halide ions which leads to the production of periodic acid with strong pro-oxidant effects. Similarly to OH, periodic acid damages proteins in intestinal villi and induces cell lysis (184). Mutual interactions between mast cells, basophils, eosinophils, and macrophages, and the secreted mediators (cytokines, chemokines, enzymes, and ROS) can damage the epithelium of intestinal villi (185). ROS exert cytotoxic effects, mainly by promoting rapid peroxidation of membrane lipids (186). Lipids are cell components that are most susceptible to oxidative modifications. Lipid oxidation products (in particular aldehydes such as MDA) can modify the biophysical properties of cell membranes. These compounds increase cell permeability and inhibit the activity of selected membrane enzymes/transport proteins, but they can also induce COX-2 expression in macrophages (87).

### The maillard reaction and food allergies

Allergens occur naturally in food products. The most common food allergens are cow’s milk, egg, and wheat proteins, fish, seafood, nuts, tomatoes, celery, spices, and food additives (such as colorants, flavor enhancers, and preservatives). These compounds can be also produced during thermal processing which not only causes structural changes in food components, but also affects interactions with other proteins, lipids, and carbohydrates (180). Numerous studies have shown that the Maillard reaction is responsible for the immunogenicity and allergenicity of food proteins (187–189). The Maillard reaction involves many chemical processes that are initiated by direct interactions between a carboxyl or hemiacetal group in a reducing sugar and an amine group in an amino acid or a peptide. The Maillard reaction leads to the formation of AGEs, and the role of oxidative and carbonyl stress in the pathogenesis of food allergies has been investigated worldwide (180).

Most food allergies are IgE-dependent (185). IgE recognizes specific structure of epitopes in the structured regions of allergenic proteins (190). Numerous studies had been undertaken to determine whether glycation of food allergens during the Maillard reaction modifies IgE reactivity and leads to the formation of new IgE epitopes (191). The results are presented in **Table 2**. It was found that the Maillard reaction can prevent IgE from binding to food allergens (192). Some individuals are allergic only to stored or cooked foods (such as processed fish and peanuts), but not to raw foods. Thermal processing induces changes in the structure of allergenic proteins (193). Food proteins are denatured under exposure to high temperatures, whereas protein refolding, oligomerization, and aggregation are observed at low temperatures. As a result, IgE reactivity may be enhanced in some allergens because some IgE epitopes become more exposed through heat treatment (185). Glycation could be the main factor that decreases the affinity and/or availability of allergens for specific IgE antibodies by modifying the electric charge, hydrophobicity, and/or structure of protein molecules. However, heat treatment can also alter the allergenicity of food proteins. An *in vitro* study demonstrated that glycation can mask IgE proteins and decrease their availability for specific IgE antibodies, which decreases a protein’s allergenicity (193). Therefore, the Maillard reaction can be helpful in reducing the allergenic potential of some thermally processed food products (194).

**Table 2:** The influence of protein glycation on IgE reactivity.

Allergen(s)	Sugar	Treatment	Influence of glycation on IgE reactivity and/or mediator release capacity of allergen	Reference
Peanut (allergens)				
Ara h 1	Glucose	heating, 15 min, 100°C	↓IgE reactivity and mediator release capacity in response to heating	Blanc F (273). (2011)
Ara h 1	Glucose	incubation, 20 min, 145°C	↓ IgE reactivity and enhanced mediator release capacity in response to heating. Significant ↓ in IgE reactivity in response to glycation	Vissers Y (274). (2011)
Ara h 1	Glucose	heating, 60 min, 100°C	Enhanced IgE reactivities of Ara h 1 and Ara h 2 in response to incubation with selected sugars	Maleki S (275). (2000)
Ara h 2	Maltose Mannose Xylose		Enhanced IgE reactivities of Ara h 1 and Ara h 2 in response to incubation with selected sugars	
Ara h 2	Glucose Fructose Maltose Ribose	heating, 100 min, 90°C	Enhanced IgE reactivity in response to incubation with ribose	Gruber P (276). (2005)
Ara h 2/6	Glucose	heating, 15 min, 110°C	↓ IgE reactivity and mediator release capacity in response to heating	Vissers Y (277). (2011)
Ara h 2/6	Glucose	incubation, 20 min, 145°C	↓ IgE reactivity and mediator release capacity in response to heating	Vissers Y (274). (2011)
Hazelnuts (allergens)				
Cor a 11	Glucose	incubation, 20 min, 145°C	↓IgE reactivity and enhanced mediator release capacity in response to heating. Glycation promoted the reduction of IgE reactivity and counteracted enhanced mediator release capacity in response to heating	Iwan M (278). (2011)
Cherry (allergens)				
Pru av 1	Glucose Fructose Maltose Ribose	heating, 30-90 min, 100°C	↓ IgE reactivity in response to incubation with ribose for 90 min	Gruber P (279). (2005)
Apples (allergens)				
Mal d 3	Glucose	boiling, 20 min at 100°C or 120 min at 90°C	↓ IgE reactivity and mediator release capacity in response to boiling at 100°C for 2 h. Glycation counteracted the boiling effect.	Sancho A (280). (2005)
Shellfish (allergens)				
Tropomyosin	Glucose Maltotriose Maltose Ribose	Incubation: 60°C for 30 min – 48 h; 60°C for 12 h-15 days; 60°C for 5-180 min	↑ IgE reactivity in response to incubation with glucose	Nakamura A (281). (2005)
Squid (allergens)				
Tropomyosin	Ribose	incubation, 180 min, 60°C	↓IgE reactivity	Nakamura A (282). (2006)
Eggs (allergens)				
Ovomucoid	Glucose	incubation, 96h, 50°C	↓IgE reactivity	Jiménez-Saiz R (283). (2011)
Milk (allergens)				
β-Lactoglobulin	Galactose Glucose Lactose Rhamnose Ribose Arabinose	heating, 72h, 60°C	↓IgE reactivity	Taheri-Kafrani A (284). (2009)

↓ means "decresed".

Recent research suggests that Maillard reaction products affect APCs (in particular macrophages and DCs) and disrupt T cell responses under exposure to an allergen. DCs express several receptors for AGEs (RAGE) (195), including galectin-3 (28), type I and II class A macrophage scavenging receptors (SR-A), type I class B scavenger receptor (SR-B), and CD36 (196). An *in vitro* study revealed that AGEs binding to RAGE on macrophages and microglia promote oxidative stress by activating NF-KB, and MAPK, c-Jun N-terminal kinase (JNK), and p21RAS signaling pathways (197). NF-KB is sensitive to ROS, and it modulates the transcription of genes encoding endothelin-1, tissue factor, and thrombomodulin (198). The above increases the production of pro-inflammatory cytokines, chemokines, and growth factors (187, 199, 200). The involvement of AGEs in food allergies is further evidenced by the fact that AGEs induce DC maturation, thus stimulating T cell responses directed against allergens (201). However, AGEs were found to both stimulate (202) and inhibit (203) APC maturation. In a study by Ge et al., AGEs of bovine serum albumin (AGE-BSA) stimulated the maturation of human DCs and increased their ability to activate T cells (204). In turn, Price et al. reported that AGEs of the adrenocorticotropic hormone (ACTH) inhibited the maturation of human DCs (201). These apparent discrepancies could be attributed to differences in the expression of AGE receptors on the surface of various cell types. In addition, glycated food allergens can increase the immunogenicity of T cells and decrease the threshold doses of food allergens that produce an adverse reaction (202). However, further research is needed to expand our knowledge about the glycation of structures that bind to different AGE receptors and the impact of AGE on DC maturation (205).

## Antioxidant therapies for allergic diseases

As previously demonstrated, enzymatic and non-enzymatic antioxidant systems are impaired in asthma, AR, AD and urticaria, which increases cell susceptibility to oxidative damage (59, 145, 171, 206). The antioxidant barrier consists of antioxidant enzymes, free radical scavengers, and preventive antioxidants (10). Antioxidants significantly decrease ROS generation, and antioxidant supplements may be effective in allergy treatment. Antioxidant vitamins (A, C, D, E) and minerals ions (iron, zinc, selenium) are also known to suppress inflammation (207, 208). Vitamin A is essential for the maturation and differentiation of lymphocytes, monocytes, neutrophils, and eosinophils. Vitamin C increases the absorption and mobilisation of non-heme iron, which is essential for the proper functioning of the immune system. Due to its reducing properties, vitamin C protects neutrophils, lymphocytes and macrophages from ROS overproduction. It also participates in the energy metabolism of the cell (209). Vitamin D has strong immunomodulatory properties. The active form of vitamin D (calcitriol) stimulates the innate response (i.e. chemotactic and phagocytotic properties), but also the production of antimicrobial peptides, i.e. cathelicidins (210). Vitamin D deficiency is associated with numerous autoimmune, infectious and cancerous diseases (211). The action of vitamin E includes scavenging of organic radicals and terminating lipid peroxidation in biological membranes. Vitamin E attenuates the synthesis of prostaglandin E2 (PGE2), thereby regulating the balance between Th1 and Th2 lymphocytes in favour of Th2 cells (209). Anti-inflammantory and antioxidant effects of vitamin A (212, 213), C (214, 215), D (216, 217) and E (213) also involve inhibition of NF-kB as well as activation of *Nrf-2* signaling pathways involved in cellular antioxidant response (218, 219). Polyunsaturated fatty acids (PUFAs) also have antioxidant and anti-inflammatory properties. PUFAs of the n-3 and n-6 series cannot be synthesised by humans and must be supplied in the diet. These include α-linolenic acid (C 18:3), which is a precursor of eicosapentaenoic acid (EPA) and docosahexaenoic acid (DHA), and linoleic acid (C 18:2), the precursor of arachidonic acid (AA) (220, 221). Also intake a flavonoids/polyphenols as antioxidants to decrease ROS generation [(218, 219)].

The role of vitamin D in the treatment of allergic diseases has not been fully elucidated (**Table 3**) (222). However, recent research suggests that vitamin D deficiency could be linked with the induction and severity of allergic asthma (223). A negative correlation was noted between serum vitamin D levels and the prevalence of bronchial asthma (224, 225). Vitamin D is an antioxidant that decreases ROS generation (222). This compound modulates macrophage and DC functions, induces CD4+ and CD25+ T cells, and stimulates the development of Th2 cells. Vitamin D also increases the number of Treg cells that inhibit allergic skin reactions, and it exerts anti-inflammatory effects by limiting the overproduction of TNF-α (226). This compound also induces the expression of thymic stromal lymphopoietin (TSLP) which participates in the induction of an AD-like phenotype (227). However, despite low vitamin D levels in asthma patients, hypovitaminosis D has not been linked with a higher risk or increased severity of asthma (228). There is no evidence to indicate that vitamin D is effective in preventing asthma or the associated complications. One study demonstrated that vitamin D deficiency plays a key role in asthma induction and that vitamin D supplements are effective in preventing or alleviating asthma symptoms (211). In another study, vitamin D supplements did not improve asthma symptoms in patients undergoing conventional therapy (229). Individual responses to vitamin D supplementation may vary, and they are determined by many factors, including baseline blood levels of vitamin D and the patient’s age. Vitamin D supplementation may be particularly effective in individuals with hypovitaminosis D. The analyzed studies investigated different vitamin D doses and supplementation periods, which could have affected the results (228). However, it should be remembered that the main source of vitamin D in the body is sun exposure, and only a small amount is carried through the diet (230).

**Table 3:** Antioxidants and their supplementation in allergic diseases.

Study group	Control group	Methods	Endpoints	Reference
Vitamin D				
80 asthmatic patients (aged 18-50)	80 healthy subjects (aged 18-50)	25-hydroxyvitamin D3 (ELISA)	No correlations between 25-hydroxyvitamin D3 levels and asthma severity	Deveruex (2010) (224)
989 children aged 6 (M/F - 554/435) and and 1380 children aged 14 - (M/F - 714/666)	No control group	25-hydroxyvitamin D3 (ELISA)	Vitamin D deficiency increases the risk of atopy, bronchial hyperresponsiveness (BHR), and asthma.	Hollams (2011) (225)
85 asthmatic patients aged 45-48 (M/F - 26/59)	73 healthy subjects aged 40-44 (M/F - 37/36)	25-hydroxyvitamin D3 (ELISA)	Vitamin D levels were lower in asthmatic patients than in the control group	Tamašauskienė (2015) (211)
30 patients with moderate asthma (vitamin D supplementation 60,000 IU/week)	30 patients with moderate asthma (without vitamin D supplementation)	25-hydroxyvitamin D3, pulmonary function tests (spirometry)	Vitamin D supplementation failed to improve lung function in adults with moderate asthma administered inhaled corticosteroids	Sharma (2017) (229)
Vitamin A				
35 asthmatic children aged 2-12 (M/F - 24/11)	29 asthmatic children aged 2-12 (M/F - 19/10)	vitamin A (HPLC)	Vitamin A levels were significantly lower in children with asthma than in controls (p<0.0001).	Arora (2002) (235)
Vitamin C				
18737 children aged 6-7		Standardized respiratory questionnaires	Consumption of fruit rich in vitamin C, even at low intake levels, may reduce wheezing in children with asthma	Forastiere (2002) (234)
62 children with AD aged 1-13 (M/F - 33/29) (individual intake was calculated)		vitamin C (HPLC)	vitamin C helps improve chronic inflammation and positively influences AD	Lim (2013) (233)
280,041 children born to pregnant smokers (vitamin C - 500 mg/day)	260,429 children born to pregnant smokers (vitamin C - 60 mg/day)	Analysis of asthma prevalence between birth and the age of 18 years	Vitamin C supplementation in pregnant smokers is a safe and inexpensive intervention that may reduce the economic burden of pediatric asthma	Yieh (2018) (285)
71 patients aged 28-60 (ascorbate 7.5 g/50 ml/day)		observational study	Intravenous high-dose vitamin C treatment reduces allergy-related symptoms	Vollbracht (2018) (286)
30 patients with chronic asthma (vitamin C - 1000 mg/day)	30 patients with chronic asthma (placebo)	standard pulmonary function test (PFT)	In group A (vitamin C - 1000 mg/day), spirometry parameters did not change after one month of treatment, indicating that vitamin C treatment had no effect on spirometry parameters.	Nadi (2012) (269)
Selenium				
25 patients with asthma aged 28-42 (M/F - 9/17)	25 healthy subjects aged 28-48 (M/F-9-16)	Selenium (spectrophotometry)	Abnormal distribution of Se may aggravate oxidative damage and inflammation, increase CD4/CD8 lymphocyte ratios, and decrease lung function in asthma	Chih-Hung Guo (2011) (39)
54 subjects with asthma aged 15-33		nutritional questionnaire	Increased Se intake in patients with asthma	Daniel Antonio de Luis (2003) (90)
46 patients with asthma aged 28-69 (M/F - 29/17)	75 healthy donors aged 45-59 (M/F - 9/17) (	Selenium (spectrophotometry)	Serum Se levels are significantly lower in asthmatic patients	Durdi Qujeq (2003) (248)
42 asthmatic children aged 2-13 (M/F - 24/20)	30 healthy children aged 2-14 (M/F - 19/13)	Selenium (spectrophotometry)	Decreased Se levels increase asthma risk	Kocyigit (2004) (287)
PUFAs				
166 children with asthma	169 healthy children	Questionnaires on dietary intake of n-3 and n-6 PUFAs	Foods rich in n-3 PUFAs and low in n-6 PUFAs deliver modulatory effects and protect children against asthma	Oddy (2004) (259)
Children of mothers with a family history of asthma receiving omega-3 supplements (500 mg/day)	Children of mothers without a family history of asthma receiving n-3 PUFA- supplements	n-3 PUFA levels	High plasma levels of n-3 PUFAs had no effect on the prevalence of diagnosed asthma or atopy	Mihrshahi (2004) (262)
393 children aged 18 months, 400 children aged 3 years, and 396 children aged 5 years (500 mg/day)		Plasma levels of n-3 and n-6 PUFAs (gas chromatography)	Plasma levels of fatty acids, dietary intake, and supplementation were not associated with any respiratory or allergic outcomes	Almqvist (2007) (261)
368 pregnant women administered 2.4 g of n-3 LCPUFA/day	368 pregnant women (placebo)	cohort study	Less persistent wheezing/asthma between the ages of 3 to 5 years	Bisgaard (2016) (265)
266 pregnant women administered 2.7 g of n-3 PUFAs/day)	136 pregnant women administered capsules with olive oil and 136 pregnant women not administered olive oil capsules	cohort study	Assuming that the administered dose of olive oil was inert, increased intake of n-3 PUFAs in late pregnancy may prevent asthma in the offspring	Olsen (2008) (266)

ELISA, enzyme-linked immunosorbent assay; HPLC, high-performance liquid chromatography; PFT, pulmonary function test; PUFAs, polyunsaturated fatty acids.

Vitamin C is a potent antioxidant. Vitamin C can donate two electrons, and it functions as a cofactor in many enzymatic reactions and participates in the regeneration of other antioxidants (231, 232). Lim et al. found that vitamin C supplementation reduced the severity of AD symptoms in children (233). Research conducted on AD and asthma patients revealed that foods rich in vitamins C and A reduce the risk of these diseases (234, 235). Interestingly, Rosenluld et al. (236) observed an inverse association between β-carotene intake and rhinitis in a study of 246 children. It was shown that vitamin C and vitamin A have immunomodulatory properties that promote a balanced immune response, reduce the production of pro-inflammatory mediators and inhibit allergic reactions (237). Importantly, vitamin A is one of the few antioxidants with the ability to remove singlet oxygen and organic lipid peroxides (238).

Selenium is an essential micronutrient very important for various aspects of human health (239). Adequate selenium levels are crucial for initiating and regulating the immune response and inhibiting chronic inflammation (240, 241). Dietary selenium, mainly through incorporation into selenoproteins (242, 243), can influence various leukocyte functions such as adhesion, migration, phagocytosis and cytokine secretion. Members of the selenoprotein family regulate or are regulated by cellular redox balance. The link between selenoproteins and Ca^2+^ flux is especially important. This compound regulates the oxidative burst that is required for optimal immune cell activation (244, 245). What is more selenium significantly influences the activation, proliferation, and differentiation of T cells during the initiation of immune response (242). In a study of mice fed diets with a low, moderate, and high content of selenium, CD4+ T cells differed in the expression of T-cell receptors (TCRs). High selenium intake enhanced T cell proliferation and promoted the differentiation of CD4^+^ T cells into Th1 (246). Moreover, selenium is a component of the active center of GPx, one of the key enzymes that reduce hydrogen peroxide in cells (242). Allergies are mediated by oxidative stress, which is why dietary selenium can play an important role in the pathogenesis of these diseases. By inhibiting GPx activity, selenium deficiency can contribute to inflammation in asthma (247). According to some researchers, selenium intake is associated with the prevalence and progression of asthma (90, 248). Selenium supplementation considerably increased selenium levels and GPx activity in the blood and significantly improved the quality of patients’ life, but it had no effect on the clinical parameters of lung function or airway hyperreactivity (246, 247). However, respiratory function was found to be impaired in asthma patients characterized by lower selenium levels than control group subjects (39). There is evidence to suggest that selenium supplementation may be helpful in standard therapy of chronic asthma. Selenium supplements may reduce glucocorticoid consumption in asthma patients (246, 249). A study conducted on an animal model revealed that similarly to vitamin D, the benefits of selenium supplementation are largely influenced by baseline selenium concentration (250). The above could be attributed to baseline T cell differentiation status, which is partly influenced by selenium (247). These results seem to confirm previous observations that selenium intake is not linked with allergic airway inflammation (246). There is no compelling evidence to indicate that dietary selenium is helpful in reducing allergic asthma (250). However, due to its antioxidant and anti-inflammatory properties, selenium can boost resistance to infections, and it may be administered as adjunct therapy in allergies (247). Studies showed that vitamins C and E promote selenium absorption. Higher serum levels of selenium have been associated with decreased prevalence of asthma (251) and increased lung function (252).

Magnesium supplementation may also be helpful in the treatment of allergic diseases. Rosenluld et al. (236) in a study of 246 children observed that magnesium intake was inversely associated with asthma and atopic sensitisation. Magnesium has been shown to stimulate complement activation and phagocytosis reducing the formation of pro-inflammatory interleukins or ROS (253). However, the exact role of magnesium in alleviating asthma complications remains unknown and requires further research.

n-3 PUFAs also modulate cellular redox homeostasis and influence the progression of allergic reactions. They have been shown to inhibit the production of IL-1, 2 and 6 and the phosphorylation of IkB kinase (IkappaB) (254), and thus the NF-kB signalling pathway. In addition, n-3 PUFAs regulate the synthesis of leukotrienes, prostaglandins and thromboxanes. As a result, n-3 PUFAs may deliver protective effects in inflammatory diseases, including asthma (255). Studies have shown that consumption of oily fish (256, 257) and n-3 PUFA supplementation reduce the incidence of asthma in children (258). In turn, high consumption of n-6 PUFAs has been linked with increased prevalence of allergies in children (259), but this observation was not confirmed by other studies (260–262). There is no conclusive evidence to indicate that n-3 PUFAs exert positive effects and n-6 PUFAs exert negative effects or that the dietary n-6:n-3 PUFA ratio influences the prevalence of asthma in adults (258). However, the moment when patients start taking supplemental n-3 PUFAs is important. Numerous studies have demonstrated that maternal nutrition affects infant health and predisposition to allergic diseases not only in childhood, but also in adulthood (263–267).

Research findings suggest that dietary antioxidants and modulators of redox homeostasis, including vitamins C and D, selenium, and n-3 PUFAs, are modifiable risk factors in the development of allergic diseases, although further clinical trials are needed to confirm this hypothesis (**Table 3**) (268). These compounds can be administered as adjuvant therapy to improve clinical outcomes in allergy patients (261, 269). However, there is no compelling evidence to indicate that antioxidants reduce the severity of allergy symptoms or slow down disease progression. These discrepancies can be attributed to differences in the patients’ dietary habits, comorbidities, antioxidant doses, and duration of therapy. In addition, recent studies indicate that some drugs routinely used in the treatment of allergies (H1 receptor antagonists) have additional anti-glycooxidant and anti-inflammatory effects, which may offset allergy complications caused by oxidative and carbonyl stress (270). Further research on the use of H1 blockers and antioxidants in the treatment of allergic diseases is needed.

## Conclusions

There is some evidence to indicate that oxidative and nitrosative stress is involved in the pathogenesis of asthma, AR, AD and urticaria. Inflammatory cells produce much larger quantities of ROS/RNS in allergic patients than in healthy subjects. Exposure to both inhalant allergens (ultrafine carbon particles, DEPs, cigarette smoke, pollen) and food allergens leads to ROS overproduction. Although the exact molecular mechanisms are not known, ROS and RNS play an important role in respiratory and skin allergies. ROS exacerbate the severity of asthma symptoms by activating inflammatory mediators that cause airway smooth muscle contraction, promote mucus hypersecretion, increase the permeability of lung capillaries, and damage cell membranes. Enzymatic and non-enzymatic antioxidant systems are impaired in allergic conditions, which increases cell susceptibility to oxidative damage. Although even low doses of antioxidants can prevent or delay the oxidation of proteins, lipids and nucleic acids, there is no convincing evidence to suggest that antioxidants reduce the severity of allergy symptoms or slow the progression of the disease. However, antioxidants and modulators of redox homeostasis could be modifiable risk factors in the onset and progression of allergies. Therefore, further basic and clinical research is needed to elucidate the role of oxidative stress in allergic diseases.

## References

[1] Lipozencić J. A hundred years of “Allergy” Clemens von Pirquet’s essay “Allergie”, published on July 24, 1906 in Münchener Medizinische Wochenschrift. Acta dermatovenerologica Croatica : ADC/Hrvatsko dermatolosko drustvo. (2006) 14:206. Available online at: https://neuro.unboundmedicine.com/medline/citation/17010271/A_hundred_years_of_”Allergy”_Clemens_von_Pirquet’s_essay_”Allergie”_published_on_July_24_1906_in_Münchener_Medizinische_Wochenschrift_. 17010271

[2] Rajan TV. The Gell-Coombs classification of hypersensitivity reactions: A re-interpretation. Trends Immunol. (2003) 24:376–9. doi: 10.1016/S1471-4906(03)00142-X 12860528

[3] Fal AM. Alergia, choroby alergiczne, astma. (2011) 2.

[4] Huber L. Style adaptacyjne do sytuacji stresowych w różnych grupach wiekowych, a choroby cywilizacyjne XXI wieku Styles of adaptative mechanisms to situations of stress among people of different age and the 21st century civilization diseases. Probl Hig Epidemiol. (2010) 91(2):268–75.

[5] Checa J Aran JM. Reactive oxygen species: drivers of physiological and pathological processes. J Inflammation Res. (2020) 13:1057–73. doi: 10.2147/JIR.S275595 PMC7719303 33293849

[6] Sharifi-Rad M v. AKN Zucca P Varoni EM Dini L Panzarini E. Lifestyle, oxidative stress, and antioxidants: back and forth in the pathophysiology of chronic diseases. Front Physiol. (2020) 11:694/BIBTEX. doi: 10.3389/FPHYS.2020.00694/BIBTEX 32714204 PMC7347016

[7] Barrera G. Oxidative stress and lipid peroxidation products in cancer progression and therapy. ISRN Oncol. (2012) 2012:1–21. doi: 10.5402/2012/137289 PMC3483701 23119185

[8] Pizzino G Irrera N Cucinotta M Pallio G Mannino F Arcoraci V. Oxidative stress: harms and benefits for human health. Oxid Med Cell Longev. (2017) 2017:1–13. doi: 10.1155/2017/8416763 PMC5551541 28819546

[9] Rog J Łobejko Ł. Michalina Hordejuk M Hordejuk W Marciniak W Derkacz R, Kiljańczyk A. Pro/antioxidant status and selenium, zinc and arsenic concentration in patients with bipolar disorder treated with lithium and valproic acid. Front Mol Neurosci. (2024) 17. doi: 10.3389/fnmol.2024.1441575 PMC11423611 39324118

[10] Birben E Sahiner UM Sackesen C Erzurum S Kalayci O. Oxidative stress and antioxidant defense. World Allergy Organ J. (2012) 5:9–19. doi: 10.1097/WOX.0B013E3182439613 23268465 PMC3488923

[11] Jopkiewicz S. Oxidative stress Part I. Oxidative stress as a factor in the development of civilization diseases. Medycyna Środowiskowa. (2018) 21:48–52. doi: 10.19243/2018207

[12] Husain S Hillmann K Hengst K Englert H. Effects of a lifestyle intervention on the biomarkers of oxidative stress in non-communicable diseases: A systematic review. Front Aging. (2023) 4:1085511. doi: 10.3389/fragi.2023.1085511 36970730 PMC10034086

[13] Seyedsadjadi N Grant R. The potential benefit of monitoring oxidative stress and inflammation in the prevention of non-communicable diseases (NCDs). Antioxidants. (2021) 10:1–33. doi: 10.3390/antiox10010015 PMC7824370 33375428

[14] Caturano A D’Angelo M Mormone A Russo V Mollica MP Salvatore T. Oxidative stress in type 2 diabetes: impacts from pathogenesis to lifestyle modifications. Curr Issues Mol Biol. (2023) 45:6651–66. doi: 10.3390/cimb45080420 PMC10453126 37623239

[15] Zuo L Prather ER Stetskiv M Garrison DE Meade JR Peace TI. Inflammaging and oxidative stress in human diseases: From molecular mechanisms to novel treatments. Int J Mol Sci. (2019) 20:1–39. doi: 10.3390/ijms20184472 PMC6769561 31510091

[16] Lu J Liu J Li A. Roles of neutrophil reactive oxygen species (ROS) generation in organ function impairment in sepsis. J Zhejiang Univ Sci B. (2022) 23:437–50. doi: 10.1631/jzus.B2101075 PMC9198233 35686524

[17] Sul OJ Ra SW. Quercetin prevents lps-induced oxidative stress and inflammation by modulating nox2/ros/nf-kb in lung epithelial cells. Molecules. (2021) 26:6949. doi: 10.3390/molecules26226949 34834040 PMC8625571

[18] Lugrin J Rosenblatt-Velin N Parapanov R Liaudet L. The role of oxidative stress during inflammatory processes. Biol Chem. (2014) 395:203–30. doi: 10.1515/HSZ-2013-0241 24127541

[19] Beckman JS Chen J Ischiropoulos H Crow JP. Oxidative chemistry of peroxynitrite. Methods Enzymol. (1994) 233:229–40. doi: 10.1016/S0076-6879(94)33026-3 8015460

[20] Martínez MC Andriantsitohaina R. Reactive nitrogen species: Molecular mechanisms and potential significance in health and disease. Antioxid Redox Signal. (2009) 11:1–10. doi: 10.1089/ars.2007.1993 19014277

[21] Bektemur G Bozali K Colak S Aktas S Guler EM. Oxidative stress and inflammation in COVID-19 patients. North Clin Istanb. (2023) 10:335–40. doi: 10.14744/nci.2022.00947 PMC10331251 37435296

[22] Pham K Parikh K Heinrich EC. Hypoxia and inflammation: insights from high-altitude physiology. Front Physiol. (2021) 12:676782. doi: 10.3389/fphys.2021.676782 34122145 PMC8188852

[23] Falfushynska HI Sokolov E Piontkivska H Sokolova IM. The role of reversible protein phosphorylation in regulation of the mitochondrial electron transport system during hypoxia and reoxygenation stress in marine bivalves. Front Mar Sci. (2020) 7:467. doi: 10.3389/fmars.2020.00467

[24] Albano GD Montalbano AM Gagliardo R Profita M. Autophagy/mitophagy in airway diseases: impact of oxidative stress on epithelial cells. Biomolecules. (2023) 13:1–21. doi: 10.3390/biom13081217 PMC10452925 37627282

[25] Nakai K Yoneda K Kubota Y. Oxidative stress in allergic and irritant dermatitis: from basic research to clinical management. Recent Pat Inflammation Allergy Drug Discovery. (2012) 6:202–9. doi: 10.2174/187221312802652839 22827837

[26] Okayama Y. Oxidative stress in allergic and inflammatory skin diseases. Curr Drug Targets Inflammation Allergy. (2005) 4:517–9. doi: 10.2174/1568010054526386 16127829

[27] Dilek F Ozceker D Ozkaya E Guler EM Yazici M Tamay Z. Elevated nitrosative stress in children with chronic spontaneous urticaria. Int Arch Allergy Immunol. (2017) 172:33–9. doi: 10.1159/000453334 28219063

[28] Rao Q Jiang X Li Y Samiwala M Labuza TP. Can glycation reduce food allergenicity? J Agric Food Chem. (2018) 66:4295–9. doi: 10.1021/ACS.JAFC.8B00660 29660289

[29] Bowler RP. Oxidative stress in the pathogenesis of asthma. Curr Allergy Asthma Rep. (2004) 4:116–22. doi: 10.1007/S11882-004-0056-7 14769260

[30] Zeman K. Immunologiczne podstawy chorób alergicznych. Available online at: https://docplayer.pl/14600098-Wst-p-jerzy-kruszewski-15-piemiennictwo-18-rozdzia-1-immunologiczne-podstawy-patogenezy-chorob-alergicznych-krzysztof-zeman.html (Accessed January 1, 2022).

[31] Quirt J Hildebrand KJ Mazza J Noya F Kim H. Asthma. Allergy Asthma Clin Immunol. (2018) 14:1–16. doi: 10.1186/S13223-018-0279-0/TABLS/7 30275843 PMC6157154

[32] Nocker RE Out TA Weller FR de Riemer MJ Jansen HM van der Zee JS. Induced sputum and bronchoalveolar lavage as tools for evaluating the effects of inhaled corticosteroids in patients with asthma. J Lab Clin Med. (2000) 136:39–49. doi: 10.1067/mlc.2000.107305 10882226

[33] Bradding P Roberts JA Britten KM Montefort S Djukanovic R Mueller R. Interleukin-4, -5, and -6 and tumor necrosis factor-alpha in normal and asthmatic airways: evidence for the human mast cell as a source of these cytokines. Am J Respir Cell Mol Biol. (1994) 10:471–80. doi: 10.1165/AJRCMB.10.5.8179909 8179909

[34] Comhair SAA Bhathena PR Dweik RA Kavuru M Erzurum SC. Rapid loss of superoxide dismutase activity during antigen-induced asthmatic response. Lancet. (2000) 355:624. doi: 10.1016/S0140-6736(99)04736-4 10696986

[35] Rokach J Khanapure SP Hwang SW Adiyaman M Lawson JA Fitzgerald GA. The isoprostanes: A perspective. Prostaglandins. (1997) 54:823–51. doi: 10.1016/S0090-6980(97)00183-4 9533180

[36] Voynow JA Kummarapurugu A. Isoprostanes and asthma. Biochim Biophys Acta. (2011) 1810:1091. doi: 10.1016/J.BBAGEN.2011.04.016 21596100 PMC3192308

[37] Proudfoot JM Murrey MW McLean S Greenland EL Barden AE Croft KD. F2-isoprostanes affect macrophage migration and CSF-1 signalling. Free Radic Biol Med. (2018) 126:142–52. doi: 10.1016/J.FREERADBIOMED.2018.08.007 30096434

[38] Heijink IH van Oosterhout AJM. Strategies for targeting T-cells in allergic diseases and asthma. Pharmacol Ther. (2006) 112:489–500. doi: 10.1016/J.PHARMTHERA.2006.05.005 16814862

[39] Guo CH Liu PJ Hsia S Chuang CJ Chen PC. Role of certain trace minerals in oxidative stress, inflammation, CD4/CD8 lymphocyte ratios and lung function in asthmatic patients. Ann Clin Biochem. (2011) 48:344–51. doi: 10.1258/ACB.2011.010266 21546427

[40] Elias JA Zhu Z Chupp G Homer RJ. Airway remodeling in asthma. J Clin Invest. (1999) 104:1001–5. doi: 10.1172/JCI8124 PMC408860 10525034

[41] Soumelis V Reche PA Kanzler H Yuan W Edward G Homey B. Human epithelial cells trigger dendritic cell mediated allergic inflammation by producing TSLP. Nat Immunol. (2002) 3:673–80. doi: 10.1038/NI805 12055625

[42] Knippertz I Hesse A Schunder T Kämpgen E Brenner MK Schuler G. Generation of human dendritic cells that simultaneously secrete IL-12 and have migratory capacity by adenoviral gene transfer of hCD40L in combination with IFN-γ. J Immunotherapy. (2009) 32:524–38. doi: 10.1097/CJI.0b013e3181a28422 19609245

[43] Liu L Poon R Chen L Frescura A-M Montuschi P Ciabattoni G. Acute effects of air pollution on pulmonary function, airway inflammation, and oxidative stress in asthmatic children. Environ Health Perspect. (2009) 117:668. doi: 10.1289/EHP11813 19440509 PMC2679614

[44] Szlagatys-Sidorkiewicz A Korzon M Renke J Popadiuk S. Pneumonologia i alergologia polska: organ Polskiego Towarzystwa Ftyzjopneumonologicznego, Polskiego Towarzystwa Alergologicznego, i Instytutu Gruzlicy i Chorob Pluc. (2005) 73(2):178–81. doi: 10.5603/ARM.28099 16756149

[45] Fitzpatrick AM Teague WG Holguin F Yeh M Brown LAS. Airway glutathione homeostasis is altered in children with severe asthma: evidence for oxidant stress. J Allergy Clin Immunol. (2009) 123:146–52. doi: 10.1016/J.JACI.2008.10.047 PMC2649685 19130935

[46] MacPherson JC Comhair SAA Erzurum SC Klein DF Lipscomb MF Kavuru MS. Eosinophils are a major source of nitric oxide-derived oxidants in severe asthma: characterization of pathways available to eosinophils for generating reactive nitrogen species. J Immunol. (2001) 166:5763–72. doi: 10.4049/JIMMUNOL.166.9.5763 11313420

[47] Pediatrii K Onkologii Dziecięcej Akademii Medycznej Gdańsku G Szlagatys-Sidorkiewicz A Góra-Gębka M Korzon M. Reaktywne formy tlenu i bariera antyoksydacyjna w astmie Reactive oxygen species and antioxidative barrier in asthma. Adv Respir Med. (2007) 75(2):158–62. doi: 10.5603/ARM.27994 17973223

[48] Marçal LE Rehder J Newburger PE Condino-Neto A. Superoxide release and cellular gluthatione peroxidase activity in leukocytes from children with persistent asthma. Braz J Med Biol Res. (2004) 37:1607–13. doi: 10.1590/S0100-879X2004001100003 15517074

[49] Henricks PAJ Nijkamp FP. Reactive oxygen species as mediators in asthma. Pulm Pharmacol Ther. (2001) 14:409–21. doi: 10.1006/PUPT.2001.0319 11782121

[50] Cross AR Segal AW. The NADPH oxidase of professional phagocytes—prototype of the NOX electron transport chain systems. Biochim Biophys Acta. (2004) 1657:1. doi: 10.1016/J.BBABIO.2004.03.008 PMC2636547 15238208

[51] Kehrer JP. The Haber-Weiss reaction and mechanisms of toxicity. Toxicology. (2000) 149:43–50. doi: 10.1016/S0300-483X(00)00231-6 10963860

[52] Dworski R Murray JJ Roberts LJ Oates JA Morrow JD Fisher L. Allergen-induced synthesis of F(2)-isoprostanes in atopic asthmatics. Evidence oxidant stress. Am J Respir Crit Care Med. (1999) 160:1947–51. doi: 10.1164/AJRCCM.160.6.9903064 10588611

[53] Boyce JA Finkelman F Shearer WT Vercelli D Ciencewicki J Trivedi S. Oxidants and the pathogenesis of lung diseases. J Allergy Clin Immunol. (2008) 122:456–68. doi: 10.1016/J.JACI.2008.08.004 PMC2693323 18774381

[54] Riedl MA Nel AE. Importance of oxidative stress in the pathogenesis and treatment of asthma. Curr Opin Allergy Clin Immunol. (2008) 8:49–56. doi: 10.1097/ACI.0B013E3282F3D913 18188018

[55] Bankers-Fulbright JL Kita H Gleich GJ O’Grady SM. Regulation of human eosinophil NADPH oxidase activity: A central role for PKCδ. J Cell Physiol. (2001) 189:306–15. doi: 10.1002/JCP.10022 11748588

[56] Nomura K Imai H Koumura T Kobayashi T Nakagawa Y. Mitochondrial phospholipid hydroperoxide glutathione peroxidase inhibits the release of cytochrome c from mitochondria by suppressing the peroxidation of cardiolipin in hypoglycaemia-induced apoptosis. Biochem J. (2000) 351:183–93. doi: 10.1042/0264-6021:3510183 PMC1221349 10998361

[57] Schock BC Young IS Brown V Fitch PS Shields MD Ennis M. Antioxidants and oxidative stress in BAL fluid of atopic asthmatic children. Pediatr Res 2003 53:3. (2003) 53:375–81. doi: 10.1203/01.pdr.0000049625.51462.d1 12595583

[58] O’Donnell RA Frew AJ. Is there more than one inflammatory phenotype in asthma? Thorax. (2002) 57:566–8. doi: 10.1136/THORAX.57.7.566 PMC1746386 12096196

[59] Nishiyama A Zanatta AL Junior PVM. The prevention of oxidative stress improve asthmatic inflammation. Adv Bioscience Biotechnol. (2012) 03:1087–90. doi: 10.4236/ABB.2012.38132

[60] Kirkham P Rahman I. Oxidative stress in asthma and COPD: antioxidants as a therapeutic strategy. Pharmacol Ther. (2006) 111:476–94. doi: 10.1016/J.PHARMTHERA.2005.10.015 16458359

[61] Cho YS Moon HB. The role of oxidative stress in the pathogenesis of asthma. Allergy Asthma Immunol Res. (2010) 2:183. doi: 10.4168/AAIR.2010.2.3.183 20592917 PMC2892050

[62] Academiae Medicae Silesiensis Praca Oryginalna A Zakład Mikrobiologii Immunologii K Zakład Biochemii K Anestezjologii oraz K Pediatrii Wydziału Lekarskiego Oddziałem Lekarsko-Dentystycznym Zabrzu K. Ocena wybranych parametrów równowagi oksydacyjno-antyoksydacyjnej u dzieci chorych na astmę oskrzelową i atopowe zapalenie skóry. Annales Academiae Medicae Silesiensis. (2012) 66:16–23.

[63] Jopkiewicz S. Oxidative stress Part I. Oxidative stress as a factor in the development of civilization diseases. Medycyna Środowiskowa. (2018) 21:48–52. doi: 10.19243/2018207

[64] Boldogh I Bacsi A Choudhury BK Dharajiya N Alam R Hazra TK. ROS generated by pollen NADPH oxidase provide a signal that augments antigen-induced allergic airway inflammation. J Clin Invest. (2005) 115:2169–79. doi: 10.1172/JCI24422 PMC1180538 16075057

[65] Haeberle HA Takizawa R Casola A Brasier AR Dieterich HJ van Rooijen N. Respiratory syncytial virus-induced activation of nuclear factor-kappaB in the lung involves alveolar macrophages and toll-like receptor 4-dependent pathways. J Infect Dis. (2002) 186:1199–206. doi: 10.1086/344644 12402188

[66] Aguilera-Aguirre L Bacsi A Saavedra-Molina A Kurosky A Sur S Boldogh I. Mitochondrial dysfunction increases allergic airway inflammation. J Immunol. (2009) 183:5379–87. doi: 10.4049/JIMMUNOL.0900228 PMC3028535 19786549

[67] Shauna K Kellett JCM. Cellular metabolism and hypoxia interfacing with allergic diseases. J Leukoc Biol. (2024) 116:335–48. doi: 10.1093/jleuko/qiae126 38843075

[68] Corcoran SE O’Neill LAJ. HIF1α and metabolic reprogramming in inflammation. J Clin Invest. (2016) 126:1–10. doi: 10.1172/JCI84431 PMC5096812 27571407

[69] O’Neill LAJ Kishton RJ Rathmell J. A guide to immunometabolism for immunologists. Nat Rev Immunol. (2016) 16:553–65. doi: 10.1038/nri.2016.70 PMC5001910 27396447

[70] Mills EL Kelly B Logan A Costa ASH Varma M Bryant CE. Succinate dehydrogenase supports metabolic repurposing of mitochondria to drive inflammatory macrophages. Cell. (2016) 167:457–70. doi: 10.1016/j.cell.2016.08.064 PMC5863951 27667687

[71] Kay AB. Allergy and allergic diseases. New Engl J Med. (2001) 344:30–7. doi: 10.1056/nejm200101043440106 11136958

[72] Dworski R. Oxidant stress in asthma. Thorax. (2000) 55 Suppl 2:1–8. doi: 10.1136/THORAX.55.SUPPL_2.S51 PMC1765968 10992559

[73] Ghosh S Janocha AJ Aronica MA Swaidani S Comhair SAA Xu W. Nitrotyrosine proteome survey in asthma identifies oxidative mechanism of catalase inactivation. J Immunol. (2006) 176:5587–97. doi: 10.4049/JIMMUNOL.176.9.5587 16622028

[74] Al-Afaleg NO Al-Senaidy A El-Ansary A. Oxidative stress and antioxidant status in Saudi asthmatic patients. Clin Biochem. (2011) 44:612–7. doi: 10.1016/J.CLINBIOCHEM.2011.01.016 21320478

[75] Bentur L Mansour Y Brik R Eizenberg Y Nagler RM. Salivary oxidative stress in children during acute asthmatic attack and during remission. Respir Med. (2006) 100:1195–201. doi: 10.1016/J.RMED.2005.10.022 16321513

[76] Wood LG Gibson PG Garg ML Blake RJ Garcia-Caraballo S. Airway and circulating levels of carotenoids in asthma and healthy controls. J Am Coll Nutr. (2005) 24:448–55. doi: 10.1080/07315724.2005.10719490 16373941

[77] Misso NLA Brooks-Wildhaber J Ray S Vally H Thompson PJ. Plasma concentrations of dietary and nondietary antioxidants are low in severe asthma. Eur Respir J. (2005) 26:257–64. doi: 10.1183/09031936.05.00006705 16055873

[78] Vural H Aksoy N Ceylan E Gencer M Ozguner F. Leukocyte oxidant and antioxidant status in asthmatic patients. Arch Med Res. (2005) 36:502–6. doi: 10.1016/J.ARCMED.2005.02.006 16099329

[79] Romieu I Sienra-Monge JJ Ramírez-Aguilar M Téllez-Rojo MM Moreno-Macías H Reyes-Ruiz NI. Antioxidant supplementation and lung functions among children with asthma exposed to high levels of air pollutants. Am J Respir Crit Care Med. (2002) 166:703–9. doi: 10.1164/RCCM.2112074 12204869

[80] Gvozdjáková A Kucharská J Bartkovjaková M Gazdíková K Gazdík F. Coenzyme Q10 supplementation reduces corticosteroids dosage in patients with bronchial asthma. Biofactors. (2005) 25:235–40. doi: 10.1002/BIOF.5520250129 16873952

[81] Kruzel ML Bacsi A Choudhury B Sur S Boldogh I. Lactoferrin decreases pollen antigen-induced allergic airway inflammation in a murine model of asthma. Immunology. (2006) 119:159. doi: 10.1111/J.1365-2567.2006.02417.X 16800860 PMC1782357

[82] Yang LL Huang MS Huang CC Wang TH Lin MC Wu CC. The association between adult asthma and superoxide dismutase and catalase gene activity. Int Arch Allergy Immunol. (2011) 156:373–80. doi: 10.1159/000324448 21829032

[83] Masini E Bani D Vannacci A Pierpaoli S Mannaioni PF Comhair SAA. Reduction of antigen-induced respiratory abnormalities and airway inflammation in sensitized Guinea pigs by a superoxide dismutase mimetic. Free Radic Biol Med. (2005) 39:520–31. doi: 10.1016/J.FREERADBIOMED.2005.04.006 16043023

[84] Anonymous. Asthma management handbook, 4th… - google scholar (1998). Available online at: https://scholar.google.pl/scholar?q=Anonymous+(1998)+Asthma+Management+Handbook,+4th+edn.,+National+Asthma+Campaign,+Melbourne,+Australia.&hl=pl&as_sdt=0&as_vis=1&oi=scholart (Accessed September 1, 2022).

[85] Wood LG Fitzgerald DA Gibson PC Cooper DM Garg ML. Lipid peroxidation as determined by plasma isoprostanes is related to disease severity in mild asthma. Lipids. (2000) 35:967–74. doi: 10.1007/S11745-000-0607-X 11026617

[86] Shahid SK Kharitonov SA Wilson NM Bush A Barnes PJ. Exhaled 8-isoprostane in childhood asthma. Respir Res. (2005) 6:1–6. doi: 10.1186/1465-9921-6-79 PMC1188076 16042771

[87] Ayala A Muñoz MF Argüelles S. Lipid peroxidation: production, metabolism, and signaling mechanisms of malondialdehyde and 4-hydroxy-2-nonenal. Oxid Med Cell Longev. (2014) 2014:1–31. doi: 10.1155/2014/360438 PMC4066722 24999379

[88] Cordiano R Di Gioacchino M Mangifesta R Panzera C Gangemi S Minciullo PL. Malondialdehyde as a potential oxidative stress marker for allergy-oriented diseases: an update. Molecules. (2023) 28:1–22. doi: 10.3390/molecules28165979 PMC10457993 37630231

[89] He L Cui X Li Z Teng Y Barkjohn KK Norris C. Malondialdehyde in nasal fluid: A biomarker for monitoring asthma control in relation to air pollution exposure. Environ Sci Technol. (2020) 54:11405–13. doi: 10.1021/ACS.EST.0C02558 32822160

[90] de Luis DA Izaola O Aller R Armentia A Cuéllar L. Antioxidant and fat intake in patients with polinic asthma. Med Clin (Barc). (2003) 121:653–4. doi: 10.1157/13054318 14642226

[91] Qu J Li Y Zhong W Gao P Hu C. Recent developments in the role of reactive oxygen species in allergic asthma. J Thorac Dis. (2017) 9:E32. doi: 10.21037/JTD.2017.01.05 28203435 PMC5303105

[92] Ricciardolo F Nijkamp F Folkerts G. Nitric oxide synthase (NOS) as therapeutic target for asthma and chronic obstructive pulmonary disease. Curr Drug Targets. (2006) 7:721–35. doi: 10.2174/138945006777435290 16787174

[93] Förstermann U Sessa WC. Nitric oxide synthases: regulation and function. Eur Heart J. (2012) 33:829–37. doi: 10.1093/EURHEARTJ/EHR304 PMC3345541 21890489

[94] Routsi C Stanopoulos I Zakynthinos E Politis P Papas V Zervakis D. Nitroglycerin can facilitate weaning of difficult-to-wean chronic obstructive pulmonary disease patients: A prospective interventional non-randomized study. Crit Care. (2010) 14:1–11. doi: 10.1186/CC9326/FIGURES/3 PMC3219995 21078149

[95] Prado CM Martins MA Tibério IFLC. Nitric oxide in asthma physiopathology. ISRN Allergy. (2011) 2011:1–13. doi: 10.5402/2011/832560 PMC3658695 23724233

[96] Ricciardolo FLM. Multiple roles of nitric oxide in the airways. Thorax. (2003) 58:175–82. doi: 10.1136/THORAX.58.2.175 PMC1746564 12554905

[97] van der Vliet A Eiserich JP Shigenaga MK Cross CE. Reactive nitrogen species and tyrosine nitration in the respiratory tract: epiphenomena or a pathobiologic mechanism of disease? Am J Respir Crit Care Med. (1999) 160:1–9. doi: 10.1164/AJRCCM.160.1.9807044 10390372

[98] Thomassen MJ Raychaudhuri B Dweik RA Farver C Buhrow L Malur A. Nitric oxide regulation of asthmatic airway inflammation with segmental allergen challenge. J Allergy Clin Immunol. (1999) 104:1174–82. doi: 10.1016/S0091-6749(99)70010-2 10588998

[99] Thomassen MJ Buhrow LT Connors MJ Kaneko FT Erzurum SC Kavuru MS. Nitric oxide inhibits inflammatory cytokine production by human alveolar macrophages. Am J Respir Cell Mol Biol. (1997) 17:279–83. doi: 10.1165/AJRCMB.17.3.2998M 9308913

[100] Liew FY Li Y Severn A Millott S Schmidt J Salter M. A possible novel pathway of regulation by murine T helper type-2 (Th2) cells of a Th1 cell activity via the modulation of the induction of nitric oxide synthase on macrophages. Eur J Immunol. (1991) 21:2489–94. doi: 10.1002/EJI.1830211027 1717284

[101] Zamora R Vodovotz Y Billiar TR. Inducible nitric oxide synthase and inflammatory diseases. Mol Med. (2000) 6:347. doi: 10.1007/bf03401781 10952018 PMC1949959

[102] Zhang Z Kolls JK Oliver P Good D Schwarzenberger PO Joshi MS. Activation of tumor necrosis factor-alpha-converting enzyme-mediated ectodomain shedding by nitric oxide. J Biol Chem. (2000) 275:15839–44. doi: 10.1074/JBC.M000604200 10747938

[103] Mahut B Delclaux C Tillie-Leblond I Gosset P Delacourt C Zerah-Lancner F. Both inflammation and remodeling influence nitric oxide output in children with refractory asthma. J Allergy Clin Immunol. (2004) 113:252–6. doi: 10.1016/J.JACI.2003.10.038 14767438

[104] van der Vliet A Eiserich JP Shigenaga MK Cross CE. Reactive nitrogen species and tyrosine nitration in the respiratory tract: epiphenomena or a pathobiologic mechanism of disease? Am J Respir Crit Care Med. (1999) 160:1–9. doi: 10.1164/AJRCCM.160.1.9807044 10390372

[105] Barnes PJ Hansel TT. Prospects for new drugs for chronic obstructive pulmonary disease. Lancet. (2004) 364:985–96. doi: 10.1016/S0140-6736(04)17025-6 15364192

[106] Hansel TT Kharitonov SA Donnelly LE Erin EM Currie MG Moore WM. A selective inhibitor of inducible nitric oxide synthase inhibits exhaled breath nitric oxide in healthy volunteers and asthmatics. FASEB J. (2003) 17:1298–300. doi: 10.1096/FJ.02-0633FJE 12738811

[107] The interaction effect of MDSC and mTOR signaling pathway by iNOS and NO in asthma . Available online at: https://www.hkjpaed.org/aspr2017/detail.asp?id=1422 (Accessed September 1, 2022).

[108] Myeloid-derived suppressor cells (MDSCs) and mechanistic target of rapamycin (mTOR) signaling pathway interact through inducible nitric oxide synthase (iNOS) and nitric oxide (NO) in asthma . Available online at: >https://pubmed.ncbi.nlm.nih.gov/31632585/ (Accessed September 1, 2022). PMC6789223 31632585

[109] Oppenheimer J Hoyte FCL Phipatanakul W Silver J Howarth P Lugogo NL. Allergic and eosinophilic asthma in the era of biomarkers and biologics: similarities, differences and misconceptions. Ann Allergy Asthma Immunol. (2022) 129:169–80. doi: 10.1016/J.ANAI.2022.02.021 35272048

[110] Miyabe Y Kobayashi Y Fukuchi M Saga A Moritoki Y Saga T. Eosinophil-mediated inflammation in the absence of eosinophilia. Asia Pac Allergy. (2021) 11:1–8. doi: 10.5415/APALLERGY.2021.11.E30 PMC8331253 34386406

[111] Beck-Ripp J Griese M Arenz S Köring C Pasqualoni B Bufler P. Changes of exhaled nitric oxide during steroid treatment of childhood asthma. Eur Respir J. (2002) 19:1015–9. doi: 10.1183/09031936.02.01582001 12108850

[112] Zanconato S Scollo M Zaramella C Landi L Zacchello F Baraldi E. Exhaled carbon monoxide levels after a course of oral prednisone in children with asthma exacerbation. J Allergy Clin Immunol. (2002) 109:440–5. doi: 10.1067/MAI.2002.121954 11897988

[113] Han M Lee D Lee SH Kim TH. Oxidative stress and antioxidant pathway in allergic rhinitis. Antioxidants. (2021) 10:1–15. doi: 10.3390/ANTIOX10081266 PMC8389336 34439514

[114] Pawankar R Mori S Ozu C Kimura S. Overview on the pathomechanisms of allergic rhinitis. Asia Pac Allergy. (2011) 1:157. doi: 10.5415/APALLERGY.2011.1.3.157 22053313 PMC3206239

[115] Borish L. Allergic rhinitis: Systemic inflammation and implications for management. J Allergy Clin Immunol. (2003) 112:1021–31. doi: 10.1016/j.jaci.2003.09.015 14657851

[116] Hong SN Rhee CS Kim JK Park SK Han DH. Clinical characteristics of asymptomatic allergen sensitization with nasal septal deviation, often misdiagnosed as allergic rhinitis. Eur Arch Oto-Rhino-Laryngology. (2021) 278:4345–51. doi: 10.1007/s00405-021-06725-5 33665723

[117] Chaaban M Corey JP. Pharmacotherapy of rhinitis and rhinosinusitis. Facial Plast Surg Clin North Am. (2012) 20:61–71. doi: 10.1016/j.fsc.2011.10.007 22099618

[118] Zalesska-Kreccicka M Kreccicki T. Zarys otorynolaryngologii : podreccznik dla studentów i lekarzy. PZWL. (1998) 1999:32.

[119] Naclerio R Ansotegui IJ Bousquet J Canonica GW D’Amato G Rosario N. International expert consensus on the management of allergic rhinitis (AR) aggravated by air pollutants: Impact of air pollution on patients with AR: Current knowledge and future strategies. World Allergy Organ J. (2020) 13:1–22. doi: 10.1016/J.WAOJOU.2020.100106 PMC7132263 32256939

[120] Riediker M Williams R Devlin R Griggs T Bromberg P. Exposure to particulate matter, volatile organic compounds, and other air pollutants inside patrol cars. Environ Sci Technol. (2003) 37:2084–93. doi: 10.1021/ES026264Y 12785511

[121] Kopp M Ulmer C Ihorst G Seydewitz H Frischer T Forster J. Upper airway inflammation in children exposed to ambient ozone and potential signs of adaptation. Eur Respir J. (1999) 14:1–7. doi: 10.1034/j.1399-3003.1999.14d22.x 10573233

[122] Iijima MK Kobayashi T. Nasal allergy-like symptoms aggravated by ozone exposure in a concentration-dependent manner in Guinea pigs. Toxicology. (2004) 199:73–83. doi: 10.1016/J.TOX.2004.01.008 15126000

[123] de Marco R Poli A Ferrari M Accordini S Giammanco G Bugiani M. The impact of climate and traffic-related NO2 on the prevalence of asthma and allergic rhinitis in Italy. Clin Exp Allergy. (2002) 32:1405–12. doi: 10.1046/J.1365-2745.2002.01466.X 12372117

[124] Rapiejko P Jurkiewicz D Pietruszewska W Zielnik-Jurkiewicz B Woroń J Lipiec A. Treatment strategy of allergic rhinitis in the face of modern world threats. Otolaryngol Pol. (2018) 72:1–12. doi: 10.5604/01.3001.0011.8057 29748453

[125] D’Amato G. Effects of climatic changes and urban air pollution on the rising trends of respiratory allergy and asthma. Multidiscip Respir Med. (2011) 6:28. doi: 10.1186/2049-6958-6-1-28 22958620 PMC3463061

[126] Traffic-related pollutants and their impact on allergic respiratory diseases . Available online at: https://pubmed.ncbi.nlm.nih.gov/20120161/ (Accessed December 30, 2021).

[127] Alessandrini F Beck-Speier I Krappmann D Weichenmeier I Takenaka S Karg E. Role of oxidative stress in ultrafine particle-induced exacerbation of allergic lung inflammation. Am J Respir Crit Care Med. (2009) 179:984–91. doi: 10.1164/RCCM.200807-1061OC 19264975

[128] Baraldo S Turato G Badin C Bazzan E Beghé B Zuin R. Neutrophilic infiltration within the airway smooth muscle in patients with COPD. Thorax. (2004) 59:308–12. doi: 10.1136/THX.2003.012146 PMC1763819 15047950

[129] D’Amato G. Effects of climatic changes and urban air pollution on the rising trends of respiratory allergy and asthma. Multidiscip Respir Med. (2011) 6:28. doi: 10.1186/2049-6958-6-1-28 22958620 PMC3463061

[130] Porebski G Wozniak M Czarnobilska E. Residential proximity to major roadways is associated with increased prevalence of allergic respiratory symptoms in children. Ann Agric Environ Med. (2014) 21:760–6. doi: 10.5604/12321966.1129929 25528916

[131] Ozbay I Kucur C Koçak FE Savran B Oghan F. Advanced oxidation protein product levels as a marker of oxidative stress in paediatric patients with chronic tonsillitis. Acta Otorhinolaryngologica Italica. (2016) 36:381. doi: 10.14639/0392-100X-897 27958598 PMC5225793

[132] Sequeira S v. RA Rao A Sequeira S Rao AV Rao A. Increased oxidative stress and altered antioxidants status in patients with chronic allergic rhinitis. Adv Bioscience Biotechnol. (2012) 3:951–6. doi: 10.4236/ABB.2012.327117

[133] Fadlullah Aksoy YSY. Serum levels of advanced oxidation protein products in response to allergen exposure in allergic rhinitis . Available online at: https://pubmed.ncbi.nlm.nih.gov/22930093/ (Accessed December 31, 2021). 22930093

[134] Sato M Fukuyama N Sakai M Nakazawa H. Increased nitric oxide in nasal lavage fluid and nitrotyrosine formation in nasal mucosa–indices for severe perennial nasal allergy. Clin Exp Allergy. (1998) 28:597–605. doi: 10.1046/J.1365-2222.1998.00270.X 9645597

[135] Kang BH Chen SS Jou LS Weng PK Wang HW. Immunolocalization of inducible nitric oxide synthase and 3-nitrotyrosine in the nasal mucosa of patients with rhinitis. Eur Arch Otorhinolaryngol. (2000) 257:242–6. doi: 10.1007/S004050050231 10923935

[136] Celik M Tuncer A Soyer OU Saçkesen C Tanju Besler H Kalayci O. Oxidative stress in the airways of children with asthma and allergic rhinitis. Pediatr Allergy Immunol. (2012) 23:556–61. doi: 10.1111/J.1399-3038.2012.01294.X 22435922

[137] Monma M Yamaya M Sekizawa K Ikeda K Suzuki N Kikuchi T. Increased carbon monoxide in exhaled air of patients with seasonal allergic rhinitis. Clin Exp Allergy. (1999) 29:1537–41. doi: 10.1046/J.1365-2222.1999.00684.X 10520083

[138] Andersson JA Uddman R Cardell LO. Increased carbon monoxide levels in the nasal airways of subjects with a history of seasonal allergic rhinitis and in patients with upper respiratory tract infection. Clin Exp Allergy. (2002) 32:224–7. doi: 10.1046/J.1365-2222.2002.00532.X 11929486

[139] The role of disturbances of lipid metabolism in pathogenesis of allergic rhinitis . Available online at: https://pubmed.ncbi.nlm.nih.gov/22334916/ (Accessed December 31, 2021).

[140] Wollenberg A Thomsen SF Lacour JP Jaumont X Lazarewicz S. Targeting immunoglobulin E in atopic dermatitis: A review of the existing evidence. World Allergy Organ J. (2021) 14:100519. doi: 10.1016/J.WAOJOU.2021.100519 33815652 PMC8005850

[141] James EA Kwok WW. Autoreactive CD4+ T cells in atopic dermatitis. J Allergy Clin Immunol. (2011) 128:100–1. doi: 10.1016/J.JACI.2011.05.005 PMC3825261 21620451

[142] Bertino L Guarneri F Cannavò SP Casciaro M Pioggia G Gangemi S. Oxidative stress and atopic dermatitis. Antioxidants. (2020) 9:1–15. doi: 10.3390/ANTIOX9030196 PMC7139929 32111015

[143] Brandt EB Sivaprasad U. Th2 Cytokines and atopic dermatitis. J Clin Cell Immunol. (2011) 2:1–13. doi: 10.4172/2155-9899.1000110 PMC3189506 21994899

[144] Pellefigues C. IgE autoreactivity in atopic dermatitis: paving the road for autoimmune diseases? Antibodies (Basel). (2020) 9:1–22. doi: 10.3390/ANTIB9030047 32911788 PMC7551081

[145] Misery L Finlay AY Martin N Boussetta S Nguyen C Myon E. Atopic dermatitis: Impact on the quality of life of patients and their partners. Dermatology. (2007) 215:123–9. doi: 10.1159/000104263 17684374

[146] Badloe FMS de Vriese S Coolens K Schmidt-Weber CB Ring J Gutermuth J. IgE autoantibodies and autoreactive T cells and their role in children and adults with atopic dermatitis. Clin Transl Allergy. (2020) 10:1–15. doi: 10.1186/S13601-020-00338-7/FIGURES/3 32774842 PMC7398196

[147] Wojciech S Leszek B. Atopowe zapalenie skóry. Alergologia Polska - Polish J Allergol. (2012) 3(1):18–28.

[148] Briganti S Picardo M. Antioxidant activity, lipid peroxidation and skin diseases. What’s new. J Eur Acad Dermatol Venereol. (2003) 17:663–9. doi: 10.1046/J.1468-3083.2003.00751.X 14761133

[149] Tsuchida K Kobayashi M. Oxidative stress in human facial skin observed by ultraweak photon emission imaging and its correlation with biophysical properties of skin. Sci Rep. (2020) 1:1–7. doi: 10.1038/s41598-020-66723-1 PMC7295759 32541901

[150] Tsukahara H Shibata R Ohshima Y Todoroki Y Sato S Ohta N. Oxidative stress and altered antioxidant defenses in children with acute exacerbation of atopic dermatitis. Life Sci. (2003) 72:2509–16. doi: 10.1016/S0024-3205(03)00145-0 12650859

[151] Horváth I Barnes PJ. Exhaled monoxides in asymptomatic atopic subjects. Clin Exp Allergy. (1999) 29:1276–80. doi: 10.1046/J.1365-2222.1999.00661.X 10469038

[152] Tsuboi H Kouda K Takeuchi H Takigawa M Masamoto Y Takeuchi M. 8-hydroxydeoxyguanosine in urine as an index of oxidative damage to DNA in the evaluation of atopic dermatitis. Br J Dermatol. (1998) 138:1033–5. doi: 10.1046/J.1365-2133.1998.02273.X 9747368

[153] Zheng G Fu Y He C. Nucleic acid oxidation in DNA damage repair and epigenetics. Chem Rev. (2014) 114:4602–20. doi: 10.1021/CR400432D PMC4002134 24580634

[154] Leveque N Robin S Muret P Mac-Mary S Makki S Humbert P. High iron and low ascorbic acid concentrations in the dermis of atopic dermatitis patients. Dermatology. (2003) 207:261–4. doi: 10.1159/000073087 14571067

[155] Niwa Y Sumi H Kawahira K Terashima T Nakamura T Akamatsu H. Protein oxidative damage in the stratum corneum: Evidence for a link between environmental oxidants and the changing prevalence and nature of atopic dermatitis in Japan. Br J Dermatol. (2003) 149:248–54. doi: 10.1046/J.1365-2133.2003.05417.X 12932228

[156] Bengtsson, Lundberg M Avila-Cariño J Jacobsson G Holmgren A Scheynius A. Thiols decrease cytokine levels and down-regulate the expression of CD30 on human allergen-specific T helper (Th) 0 and Th2 cells. Clin Exp Immunol. (2001) 123:350–60. doi: 10.1046/J.1365-2249.2001.01453.X PMC1906006 11298119

[157] Wu Z,M MacPhee I,G Oliveira D. Reactive oxygen species in the initiation of IL-4 driven autoimmunity as a potential therapeutic target. Curr Pharm Des. (2004) 10:899–913. doi: 10.2174/1381612043452875 15032693

[158] Jeannin P Delneste Y Lecoanet-Henchoz S Gauchat JF Life P Holmes D. Thiols decrease human interleukin (IL) 4 production and IL-4-induced immunoglobulin synthesis. J Exp Med. (1995) 182:1785–92. doi: 10.1084/JEM.182.6.1785 PMC2192261 7500023

[159] Kubo M Kambayashi Y Takemoto K Okuda J Muto M Ogino K. Reactive nitrogen species formation in eosinophils and imbalance in nitric oxide metabolism are involved in atopic dermatitis-like skin lesions in NC/Nga mice. Free Radic Res. (2005) 39:719–27. doi: 10.1080/10715760500139260 16036351

[160] Vestergaard C Deleuran M. Chronic spontaneous urticaria: latest developments in aetiology, diagnosis and therapy. Ther Adv Chronic Dis. (2015) 6:304. doi: 10.1177/2040622315603951 26568807 PMC4622315

[161] Siebenhaar F Melde A Magerl M Zuberbier T Church MK Maurer M. Histamine intolerance in patients with chronic spontaneous urticaria. J Eur Acad Dermatol Venereol. (2016) 30:1774–7. doi: 10.1111/JDV.13778 27329741

[162] Jain S. Pathogenesis of chronic urticaria: an overview. Dermatol Res Pract. (2014) 2014:1–10. doi: 10.1155/2014/674709 PMC4120476 25120565

[163] Nakai K Tsuruta D. What are reactive oxygen species, free radicals, and oxidative stress in skin diseases? Int J Mol Sci. (2021) 22. doi: 10.3390/IJMS221910799 PMC8509443 34639139

[164] Raho G Cassano N D’Argento V Vena GA Zanotti F. Over-expression of Mn-superoxide dismutase as a marker of oxidative stress in lesional skin of chronic idiopathic urticaria. Clin Exp Dermatol. (2003) 28:318–20. doi: 10.1046/J.1365-2230.2003.01264.X 12780723

[165] Rajappa M Chandrashekar L Sundar I Munisamy M Ananthanarayanan PH Thappa DM. Platelet oxidative stress and systemic inflammation in chronic spontaneous urticaria. Clin Chem Lab Med. (2013) 51:1789–94. doi: 10.1515/CCLM-2012-0897/MACHINEREADABLECITATION/RIS 23612662

[166] Sly LM Kalesnikoff J Lam V Wong D Song C Omeis S. IgE-induced mast cell survival requires the prolonged generation of reactive oxygen species. J Immunol. (2008) 181:3850. doi: 10.4049/JIMMUNOL.181.6.3850 18768839 PMC2556878

[167] Nettis E Distaso M Saitta S Casciaro M Cristani M Saija A. Involvement of new oxidative stress markers in chronic spontaneous urticaria. Adv Dermatol Allergology/Postępy Dermatologii i Alergologii. (2017) 34:448–52. doi: 10.5114/ADA.2017.71110 PMC5831279 29507559

[168] Dalle-Donne I Aldini G Carini M Colombo R Rossi R Milzani A. Protein carbonylation, cellular dysfunction, and disease progression. J Cell Mol Med. (2006) 10:389. doi: 10.1111/J.1582-4934.2006.TB00407.X 16796807 PMC3933129

[169] Vistoli G de Maddis D Cipak A Zarkovic N Carini M Aldini G. Advanced glycoxidation and lipoxidation end products (AGEs and ALEs): an overview of their mechanisms of formation. Free Radic Res. (2013) 47 Suppl 1:3–27. doi: 10.3109/10715762.2013.815348 23767955

[170] Sagdic A Sener O Bulucu F Karadurmus N Yamanel L Tasci C. Oxidative stress status in patients with chronic idiopathic urticaria. Allergol Immunopathol (Madr). (2011) 39:150–3. doi: 10.1016/J.ALLER.2010.06.012 21236552

[171] Briganti S Cristaudo A D’Argento V Cassano N Turbino L Guarrera M. Oxidative stress in physical urticarias. Clin Exp Dermatol. (2001) 26:284–8. doi: 10.1046/J.1365-2230.2001.00817.X 11422177

[172] Bonetta R. Potential therapeutic applications of mnSODs and SOD-mimetics. Chem - A Eur J. (2018) 24:5032–41. doi: 10.1002/chem.201704561 29131419

[173] Czernecki T. Mechanizm powstawania IgE-zależnej alergii pokarmowej(2012). Available online at: http://www.nutrilife.pl/index.php?art=17 (Accessed December 30, 2021).

[174] Vickery BP Chin S Burks AW. Pathophysiology of food allergy. Pediatr Clin North Am. (2011) 58:363–76. doi: 10.1016/J.PCL.2011.02.012 PMC3070117 21453807

[175] Besler M Steinhart H Paschke A. Stability of food allergens and allergenicity of processed foods. J Chromatogr B BioMed Sci Appl. (2001) 756:207–28. doi: 10.1016/S0378-4347(01)00110-4 11419714

[176] Vighi G Marcucci F Sensi L di Cara G Frati F. Allergy and the gastrointestinal system. Clin Exp Immunol. (2008) 153 Suppl 1:3–6. doi: 10.1111/J.1365-2249.2008.03713.X 18721321 PMC2515351

[177] Czernecki T Targoński Z. Alergeny i alergie pokarmowe. Żywność Nauka Technologia Jakość. (2002) 09:19–33.

[178] Gupta RS Singh AM Walkner M Caruso D Bryce PJ Wang X. Hygiene factors associated with childhood food allergy and asthma. Allergy Asthma Proc. (2016) 37:e140. doi: 10.2500/AAP.2016.37.3988 27931290 PMC5080537

[179] Satitsuksanoa P Jansen K Głobińska A van de Veen W Akdis M. Regulatory immune mechanisms in tolerance to food allergy. Front Immunol. (2018) 9:2939/BIBTEX. doi: 10.3389/FIMMU.2018.02939/BIBTEX 30619299 PMC6299021

[180] Valenta R Hochwallner H Linhart B Pahr S. Food allergies: the basics. Gastroenterology. (2015) 148:1120. doi: 10.1053/J.GASTRO.2015.02.006 25680669 PMC4414527

[181] Kanagaratham C el Ansari YS Lewis OL Oettgen HC. IgE and igG antibodies as regulators of mast cell and basophil functions in food allergy. Front Immunol. (2020) 11:603050/BIBTEX. doi: 10.3389/FIMMU.2020.603050/BIBTEX 33362785 PMC7759531

[182] Nakano N Kitaura J. Mucosal mast cells as key effector cells in food allergies. Cells. (2022) 11:1–32. doi: 10.3390/CELLS11030329 PMC8834119 35159139

[183] Wolszczak-Biedrzycka B Dorf J Wojewódzka-żelezniakowicz M Żendzian-Piotrowska M Dymicka-Piekarska VJ Matowicka-Karna J. Unveiling COVID-19 secrets: harnessing cytokines as powerful biomarkers for diagnosis and predicting severity. J Inflammation Res. (2023) 16:6055–70. doi: 10.2147/JIR.S439217 PMC10723593 38107380

[184] Reis AC Alessandri AL Athayde RM Perez DA Vago JP ÁvilaT V. Induction of eosinophil apoptosis by hydrogen peroxide promotes the resolution of allergic inflammation. Cell Death Dis. (2015) 6:e1632–2. doi: 10.1038/CDDIS.2014.580 PMC4669804 25675292

[185] Anvari S Miller J Yeh CY Davis CM. IgE-mediated food allergy. Clin Rev Allergy Immunol. (2019) 57:244–60. doi: 10.1007/S12016-018-8710-3 30370459

[186] Bublin M Eiwegger T Breiteneder H. Do lipids influence the allergic sensitization process? J Allergy Clin Immunol. (2014) 134:521. doi: 10.1016/J.JACI.2014.04.015 24880633 PMC4151997

[187] Zuidmeer L Goldhahn K Rona RJ Gislason D Madsen C Summers C. The prevalence of plant food allergies: a systematic review. J Allergy Clin Immunol. (2008) 121:1210–8. doi: 10.1016/J.JACI.2008.02.019 18378288

[188] Sicherer SH Sampson HA. Food allergy: A review and update on epidemiology, pathogenesis, diagnosis, prevention, and management. J Allergy Clin Immunol. (2018) 141:41–58. doi: 10.1016/J.JACI.2017.11.003 29157945

[189] Rona RJ Keil T Summers C Gislason D Zuidmeer L Sodergren E. The prevalence of food allergy: a meta-analysis. J Allergy Clin Immunol. (2007) 120:638–46. doi: 10.1016/J.JACI.2007.05.026 17628647

[190] Dall’Antonia F Pavkov-Keller T Zangger K Keller W. Structure of allergens and structure based epitope predictions. Methods. (2014) 66:3–21. doi: 10.1016/j.ymeth.2013.07.024 PMC3969231 23891546

[191] Malanin K Lundberg M Johansson SGO. Anaphylactic reaction caused by neoallergens in heated pecan nut. Allergy. (1995) 50:988–91. doi: 10.1111/J.1398-9995.1995.TB02513.X 8834830

[192] Berrens L. Neoallergens in heated pecan nut: products of Maillard-type degradation? Allergy. (1996) 51:277–8. doi: 10.1111/J.1398-9995.1996.TB04610.X 8792931

[193] Netting M Makrides M Gold M Quinn P Penttila I. Heated allergens and induction of tolerance in food allergic children. Nutrients. (2013) 5:2028. doi: 10.3390/NU5062028 23739144 PMC3725491

[194] Toda M Heilmann M Ilchmann A Vieths S. The Maillard reaction and food allergies: Is there a link? Clin Chem Lab Med. (2014) 52:61–7. doi: 10.1515/CCLM-2012-0830/HTML 23492561

[195] Schmidt AM Yan S Brett J Mora R Nowygrod R Stern D. Regulation of human mononuclear phagocyte migration by cell surface- binding proteins for advanced glycation end products. J Clin Invest. (1993) 91:2155–68. doi: 10.1172/JCI116442 PMC288218 8387541

[196] Ohgami N Nagai R Miyazaki A Ikemoto M Arai H Horiuchi S. Scavenger receptor class B type I-mediated reverse cholesterol transport is inhibited by advanced glycation end products. J Biol Chem. (2001) 276:13348–55. doi: 10.1074/JBC.M011613200 11278947

[197] Wang Y Zhang P Zhang J Hong T. Bisdemethoxycurcumin attenuates OVA-induced food allergy by inhibiting the MAPK and NF-κB signaling pathways. Exp Ther Med. (2022) 23:1–14. doi: 10.3892/ETM.2022.11328 PMC9115631 35619631

[198] Dorrington MG Fraser IDC. NF-κB signaling in macrophages: Dynamics, crosstalk, and signal integration. Front Immunol. (2019) 10:705/BIBTEX. doi: 10.3389/FIMMU.2019.00705/BIBTEX 31024544 PMC6465568

[199] Henle T. Protein-bound advanced glycation endproducts (AGEs) as bioactive amino acid derivatives in foods. Amino Acids. (2005) 29:313–22. doi: 10.1007/S00726-005-0200-2 15997413

[200] Wolszczak-Biedrzycka B Dorf J Wojewódzka-Żelezniakowicz M Żendzian-Piotrowska M Dymicka-Piekarska V Matowicka-Karna J. Changes in chemokine and growth factor levels may be useful biomarkers for monitoring disease severity in COVID-19 patients; a pilot study. Front Immunol. 14. doi: 10.3389/fimmu.2023.1320362 PMC10794366 38239363

[201] Price CL Sharp PS North ME Rainbow SJ Knight SC. Advanced glycation end products modulate the maturation and function of peripheral blood dendritic cells. Diabetes. (2004) 53:1452–8. doi: 10.2337/DIABETES.53.6.1452 15161748

[202] Chin S Vickery BP. Pathogenesis of food allergy in the pediatric patient. Curr Allergy Asthma Rep. (2012) 12:621. doi: 10.1007/S11882-012-0296-X 22933136 PMC3493697

[203] Briceno Noriega D Zenker HE Croes CA Ewaz A Ruinemans-Koerts J Savelkoul HFJ. Receptor mediated effects of advanced glycation end products (AGEs) on innate and adaptative immunity: relevance for food allergy. Nutrients. (2022) 14:371. doi: 10.3390/NU14020371 35057553 PMC8778532

[204] Ge J Jia Q Liang C Luo Y Huang D Sun A. Advanced glycosylation end products might promote atherosclerosis through inducing the immune maturation of dendritic cells. Arterioscler Thromb Vasc Biol. (2005) 25:2157–63. doi: 10.1161/01.ATV.0000181744.58265.63 16100036

[205] Sakasai-Sakai A Takata T Suzuki H Maruyama I Motomiya Y Takeuchi M. Immunological evidence for in *vivo* production of novel advanced glycation end-products from 1,5-anhydro-D-fructose, a glycogen metabolite. Sci Rep 2019 9:1. (2019) 9:1–9. doi: 10.1038/s41598-019-46333-2 PMC6629992 31308400

[206] Han M Lee D Lee SH Kim TH. Oxidative stress and antioxidant pathway in allergic rhinitis. Antioxidants (Basel). (2021) 10:1–15. doi: 10.3390/ANTIOX10081266 PMC8389336 34439514

[207] Roth-Walter F Berni Canani R O’Mahony L Peroni D Sokolowska M Vassilopoulou E. Nutrition in chronic inflammatory conditions: Bypassing the mucosal block for micronutrients. Allergy. (2024) 79:353–83. doi: 10.1111/ALL.15972 38084827

[208] Roth-Walter F Adcock IM Benito-Villalvilla C Bianchini R Bjermer L Caramori G. Metabolic pathways in immune senescence and inflammaging: Novel therapeutic strategy for chronic inflammatory lung diseases. An EAACI position paper from the Task Force for Immunopharmacology. Allergy. (2024) 79:1089–122. doi: 10.1111/ALL.15977 PMC11497319 38108546

[209] Wilkinson M Hart A Milan SJ Sugumar K. Vitamins C and E for asthma and exercise-induced bronchoconstriction. Cochrane Database Systematic Rev. (2014) 2014:1–14. doi: 10.1002/14651858.CD010749.PUB2/MEDIA/CDSR/CD010749/IMAGE_N/NCD010749-AFIG-FIG03.PNG PMC6513032 24936673

[210] Al-Daragi FZ Al-Ghurabi BH. Effect of vitamin D on antimicrobial peptides levels in patients with periodontitis. Int J Health Sci (Qassim). (2022) 6(S5):8297–305. doi: 10.53730/ijhs.v6ns5.10545

[211] Tamašauskiene L Gasiuniene E Lavinskiene S Sakalauskas R Šitkauskiene B. Evaluation of vitamin D levels in allergic and non-allergic asthma. Medicina (Kaunas). (2015) 51:321–7. doi: 10.1016/J.MEDICI.2015.11.003 26739673

[212] De Bittencourt Pasquali MA Gelain DP Zeidán-Chuliá F Pires AS Gasparotto J Terra SR. Vitamin A (retinol) downregulates the receptor for advanced glycation endproducts (RAGE) by oxidant-dependent activation of p38 MAPK and NF-kB in human lung cancer A549 cells. Cell Signal. (2013) 25:939–54. doi: 10.1016/j.cellsig.2013.01.013 23333461

[213] Iddir M Brito A Dingeo G Del Campo SSF Samouda H La Frano MR. Strengthening the immune system and reducing inflammation and oxidative stress through diet and nutrition: Considerations during the covid-19 crisis. Nutrients. (2020) 12:1–39. doi: 10.3390/nu12061562 PMC7352291 32471251

[214] Morante-Palacios O Godoy-Tena G Calafell-Segura J Ciudad L Martínez-Cáceres EM Sardina JL. Vitamin C enhances NF-κB-driven epigenomic reprogramming and boosts the immunogenic properties of dendritic cells. Nucleic Acids Res. (2022) 50:10981–94. doi: 10.1093/nar/gkac941 PMC9638940 36305821

[215] Maphetu N Unuofin JO Masuku NP Olisah C Lebelo SL. Medicinal uses, pharmacological activities, phytochemistry, and the molecular mechanisms of Punica granatum L. (pomegranate) plant extracts: A review. Biomedicine Pharmacotherapy. (2022) 153:1–23. doi: 10.1016/j.biopha.2022.113256 36076615

[216] Ilchovska D Barrow DM. An Overview of the NF-kB mechanism of pathophysiology in rheumatoid arthritis, investigation of the NF-kB ligand RANKL and related nutritional interventions. Autoimmun Rev. (2021) 20:1–6. doi: 10.1016/j.autrev.2020.102741 33340772

[217] Ali A Shah SA Zaman N Uddin MN Khan W Ali A. Vitamin D exerts neuroprotection via SIRT1/nrf-2/NF-kB signaling pathways against D-galactose-induced memory impairment in adult mice. Neurochem Int. (2021) 142:1–8. doi: 10.1016/j.neuint.2020.104893 33159979

[218] Hosseini SA Shateri Z Abolnezhadian F Maraghi E Haddadzadeh Shoushtari M Zilaee M. Does pomegranate extract supplementation improve the clinical symptoms of patients with allergic asthma? A double-blind, randomized, placebo-controlled trial. Front Pharmacol. (2023) 14:1109966. doi: 10.3389/fphar.2023.1109966 36762119 PMC9905411

[219] Magrone T Jirillo E Magrone M Russo MA Romita P Massari F. Red grape polyphenol oral administration improves immune response in women affected by nickel-mediated allergic contact dermatitis. Endocr Metab Immune Disord Drug Targets. (2020) 21:1–8. doi: 10.2174/1871530320666200313152648 32167433

[220] Kelley DS Hubbard NE Erickson KL. Regulation of human immune and inflammatory responses by dietary fatty acids. Adv Food Nutr Res. (2005) 50:101–38. doi: 10.1016/S1043-4526(05)50004-4 16263429

[221] Singer P Berger I Moritz V Förster D Taube C. N-6 and N-3 PUFA in liver lipids, thromboxane formation and blood pressure from SHR during diets supplemented with evening primrose, sunflowerseed or fish oil. Prostaglandins Leukot Essent Fatty Acids. (1990) 39:207–11. doi: 10.1016/0952-3278(90)90073-T 2336450

[222] Tagliaferri S Porri D de Giuseppe R Manuelli M Alessio F Cena H. The controversial role of vitamin D as an antioxidant: results from randomised controlled trials. Nutr Res Rev. (2019) 32:99–105. doi: 10.1017/S0954422418000197 30326975

[223] Baeke F Takiishi T Korf H Gysemans C Mathieu C. Vitamin D: modulator of the immune system. Curr Opin Pharmacol. (2010) 10:482–96. doi: 10.1016/J.COPH.2010.04.001 20427238

[224] Devereux G Wilson A Avenell A McNeill G Fraser WD. A case-control study of vitamin D status and asthma in adults. Allergy. (2010) 65:666–7. doi: 10.1111/J.1398-9995.2009.02220.X 19845573

[225] Hollams EM Hart PH Holt BJ Serralha M Parsons F de Klerk NH. Vitamin D and atopy and asthma phenotypes in children: a longitudinal cohort study. Eur Respir J. (2011) 38:1320–7. doi: 10.1183/09031936.00029011 21565922

[226] Fisher SA Rahimzadeh M Brierley C Gration B Doree C Kimber CE. The role of vitamin D in increasing circulating T regulatory cell numbers and modulating T regulatory cell phenotypes in patients with inflammatory disease or in healthy volunteers: A systematic review. PloS One. (2019) 14:1–18. doi: 10.1371/JOURNAL.PONE.0222313 PMC6759203 31550254

[227] Landheer J Giovannone B Sadekova S Tjabringa S Hofstra C Dechering K. TSLP is differentially regulated by vitamin D3 and cytokines in human skin. Immun Inflammation Dis. (2015) 3:32. doi: 10.1002/IID3.48 PMC4386913 25866638

[228] Jolliffe DA Greenberg L Hooper RL Griffiths CJ Camargo CA Kerley CP. Vitamin D supplementation to prevent asthma exacerbations: a systematic review and meta-analysis of individual participant data. Lancet Respir Med. (2017) 5:881–90. doi: 10.1016/S2213-2600(17)30306-5 PMC5693329 28986128

[229] Sharma R Kumar S Dhasmana D Kalra J. Effect of vitamin D supplementation in patients of moderate asthma undergoing treatment with inhaled corticosteroids. Br J Pharm Res. (2017) 16:1–9. doi: 10.9734/BJPR/2017/32843

[230] Peroni DG Hufnagl K Comberiati P Roth-Walter F. Lack of iron, zinc, and vitamins as a contributor to the etiology of atopic diseases. Front Nutr. (2023) 9:1032481. doi: 10.3389/fnut.2022.1032481 36698466 PMC9869175

[231] Traber MG Stevens JF. Vitamins C and E: beneficial effects from a mechanistic perspective. Free Radic Biol Med. (2011) 51:1000–13. doi: 10.1016/J.FREERADBIOMED.2011.05.017 PMC3156342 21664268

[232] Ghalibaf MHE Kianian F Beigoli S Behrouz S Marefati N Boskabady M. The effects of vitamin C on respiratory, allergic and immunological diseases: an experimental and clinical-based review. Inflammopharmacology. (2023) 31:653–72. doi: 10.1007/s10787-023-01169-1 PMC9970132 36849854

[233] Lim H Song K Kim R Sim J Park E Ahn K. Nutrient intake and food restriction in children with atopic dermatitis. Clin Nutr Res. (2013) 2:52. doi: 10.7762/CNR.2013.2.1.52 23429834 PMC3572819

[234] Forastiere F Pistelli R Sestini P Fortes C Renzoni E Rusconi F. Consumption of fresh fruit rich in vitamin C and wheezing symptoms in children. SIDRIA Collaborative Group, Italy (Italian Studies on Respiratory Disorders in Children and the Environment). Thorax. (2000) 55:283–8. doi: 10.1136/THORAX.55.4.283 PMC1745721 10722767

[235] Arora P Kumar V Batra S. Vitamin A status in children with asthma. Pediatr Allergy Immunol. (2002) 13:223–6. doi: 10.1034/J.1399-3038.2002.00010.X 12144646

[236] Rosenlund H Magnusson J Kull I Håkansson N Wolk A Pershagen G. Antioxidant intake and allergic disease in children. Clin Exp Allergy. (2012) 42:1–7. doi: 10.1111/j.1365-2222.2012.04053.x 22994346

[237] Spoelstra-De Man AME Elbers PWG Oudemans-Van Straaten HM. Vitamin C: Should we supplement? Curr Opin Crit Care. (2018) 24(4):248–55. doi: 10.1097/MCC.0000000000000510 PMC6039380 29864039

[238] Motkowski R Maciejczyk M Hryniewicka M Karpińska J Mikołuć B. Effect of statin therapy on the plasma concentrations of retinol, alpha-tocopherol and coenzyme Q10 in children with familial hypercholesterolemia. Cardiovasc Drugs Ther. (2022) 36:75–84. doi: 10.1007/s10557-020-07091-w PMC8770382 33052507

[239] Gouni-Berthold I Michalke B Krone W Guallar E Berthold H. Serum selenium concentrations and hypertension in the german population. Endocr Rev. (2012) 33:1–16. doi: 10.1097/HJH.0b013e32835efecb

[240] Vaivode I Zake T Strele I Upmale-Engela S Gogins D Gersone G. Stress-related immune response and selenium status in autoimmune thyroid disease patients. Int J Mol Sci. (2023) 24:1–12. doi: 10.3390/ijms24032440 PMC9917185 36768762

[241] Sakazaki F Arakawa T Shimizu R Ogino H Okuno T Ueno H. Allergies are aggravated by mild selenium deficiency and abrogated by supplementation with selenomethionine. Food Agric Immunol. (2014) 25:477–85. doi: 10.1080/09540105.2013.837866

[242] Brigelius-Flohé R Müller C Menard J Florian S Schmehl K Wingler K. Functions of GI-GPx: lessons from selenium-dependent expression and intracellular localization. Biofactors. (2001) 14:101–6. doi: 10.1002/BIOF.5520140114 11568446

[243] Hoffmann PR Berry MJ. The influence of selenium on immune responses. Mol Nutr Food Res. (2008) 52:1–14. doi: 10.1002/mnfr.200700330 PMC3723386 18384097

[244] Proietti P Marinucci MT Del Pino AM D’Amato R Regni L Acuti G. Selenium maintains Ca2+ homeostasis in sheep lymphocytes challenged by oxidative stress. PloS One. (2018) 13:1–11. doi: 10.1371/journal.pone.0201523 PMC6066243 30059547

[245] Yao H Fan R Zhao X Zhao W Liu W Yang J. Selenoprotein W redox-regulated Ca2+ channels correlate with selenium deficiency-induced muscles Ca2+ leak. Oncotarget. (2016) 7:1–15. doi: 10.18632/oncotarget.11459 PMC5295377 27557522

[246] Allam MF Lucena RA. Selenium supplementation for asthma. Cochrane Database Syst Rev. (2004) (2):CD003538. doi: 10.1002/14651858.CD003538.PUB2 15106206 PMC9007145

[247] Norton RL Hoffmann PR. Selenium and asthma. Mol Aspects Med. (2012) 33:98–106. doi: 10.1016/J.MAM.2011.10.003 22024250 PMC3246085

[248] Qujeq D Hidari B Bijani K Shirdel H. Glutathione peroxidase activity and serum selenium concentration in intrinsic asthmatic patients. Clin Chem Lab Med. (2003) 41:200–2. doi: 10.1515/CCLM.2003.032 12667007

[249] Decreased consumption of corticosteroids after selenium supplementation in corticoid-dependent asthmatics . Available online at: https://pubmed.ncbi.nlm.nih.gov/12061082/ (Accessed December 30, 2021). 12061082

[250] Hoffmann PR Jourdan-Le Saux C Hoffmann FW Chang PS Bollt O He Q. A role for dietary selenium and selenoproteins in allergic airway inflammation. J Immunol. (2007) 179:3258–67. doi: 10.4049/JIMMUNOL.179.5.3258 17709542

[251] Zweiman B. Relationship of serum antioxidants to asthma prevalence in youth. Am J Respir Crit Care Med. (2004) 169114:393–8. doi: 10.1016/j.jaci.2004.06.007 14630617

[252] Guizhou H Cassano PA. Antioxidant nutrients and pulmonary function: The Third National Health and Nutrition Examination Survey (NHANES III). Am J Epidemiol. (2000) 151:975–81. doi: 10.1093/oxfordjournals.aje.a010141 10853636

[253] Ashique S Kumar S Hussain A Mishra N Garg A Gowda BHJ. A narrative review on the role of magnesium in immune regulation, inflammation, infectious diseases, and cancer. J Health Popul Nutr. (2023) 42:1–14. doi: 10.1186/s41043-023-00423-0 PMC10375690 37501216

[254] Maruyama H Takahashi M Sekimoto T Shimada T Yokosuka O. Linoleate appears to protect against palmitate-induced inflammation in Huh7 cells. Lipids Health Dis. (2014) 13:1–8. doi: 10.1186/1476-511X-13-78 PMC4038110 24885871

[255] Simopoulos AP. Omega-3 fatty acids in inflammation and autoimmune diseases. J Am Coll Nutr. (2002) 21:495–505. doi: 10.1080/07315724.2002.10719248 12480795

[256] Willers SM Devereux G Craig LCA McNeill G Wijga AH Abou El-Magd W. Maternal food consumption during pregnancy and asthma, respiratory and atopic symptoms in 5-year-old children. Thorax. (2007) 62:1–11. doi: 10.1136/thx.2006.074187 PMC2117307 17389754

[257] Maslova E Strom M Oken E Campos H Lange C Gold D. Fish intake during pregnancy and the risk of child asthma and allergic rhinitis - Longitudinal evidence from the Danish National Birth Cohort. Br J Nutr. (2013) 110:1313–25. doi: 10.1017/S000711451300038X PMC4035354 23473120

[258] Yang H Xun P He K. Fish and fish oil intake in relation to risk of asthma: a systematic review and meta-analysis. PloS One. (2013) 8:1–17. doi: 10.1371/JOURNAL.PONE.0080048 PMC3827145 24265794

[259] Oddy WH de Klerk NH Kendall GE Mihrshahi S Peat JK. Ratio of omega-6 to omega-3 fatty acids and childhood asthma. J Asthma. (2004) 41:319–26. doi: 10.1081/JAS-120026089 15260465

[260] Muley P Shah M Muley A. Omega-3 fatty acids supplementation in children to prevent asthma: is it worthy?-A systematic review and meta-analysis. J Allergy (Cairo). (2015) 2015:1–7. doi: 10.1155/2015/312052 PMC4556859 26357518

[261] Almqvist C Garden F Xuan W Mihrshahi S Leeder SR Oddy W. Omega-3 and omega-6 fatty acid exposure from early life does not affect atopy and asthma at age 5 years. J Allergy Clin Immunol. (2007) 119:1438–44. doi: 10.1016/J.JACI.2007.01.046 17379291

[262] Mihrshahi S Peat JK Webb K Oddy W Marks GB Mellis CM. Effect of omega-3 fatty acid concentrations in plasma on symptoms of asthma at 18 months of age. Pediatr Allergy Immunol. (2004) 15:517–22. doi: 10.1111/J.1399-3038.2004.00187.X 15610365

[263] Miles EA Calder PC. Can early omega-3 fatty acid exposure reduce risk of childhood allergic disease? Nutrients. (2017) 9:1–16. doi: 10.3390/NU9070784 PMC5537898 28754005

[264] Kim JH Ellwood PE Asher MI. Diet and asthma: looking back, moving forward. Respir Res. (2009) 10:1–7. doi: 10.1186/1465-9921-10-49 PMC2703624 19519921

[265] Bisgaard H Stokholm J Chawes BL Vissing NH Bjarnadóttir E Schoos AMM. Fish oil-derived fatty acids in pregnancy and wheeze and asthma in offspring. N Engl J Med. (2016) 375:81. doi: 10.1056/NEJMOA1503734 28029926

[266] Olsen SF Østerdal ML Salvig JD Mortensen LM Rytter D Secher NJ. Fish oil intake compared with olive oil intake in late pregnancy and asthma in the offspring: 16 y of registry-based follow-up from a randomized controlled trial. Am J Clin Nutr. (2008) 88:167–75. doi: 10.1093/AJCN/88.1.167 18614738

[267] Sordillo JE Rifas-Shiman SL Switkowski K Coull B Gibson H Rice M. Prenatal oxidative balance and risk of asthma and allergic disease in adolescence. J Allergy Clin Immunol. (2019) 144:1534–41. doi: 10.1016/j.jaci.2019.07.044 PMC6900442 31437488

[268] Halken S. Prevention of allergic disease in childhood: Clinical and epidemiological aspects of primary and secondary allergy prevention. Pediatr Allergy Immunology Supplement. (2004) 15:9–32. doi: 10.1111/j.1399-3038.2004.0148b.x 15125698

[269] Nadi E Tavakoli F Zeraati F Goodarzi MT Hashemi SH. Effect of vitamin C administration on leukocyte vitamin C level and severity of bronchial asthma. Acta Med Iran. (2012) 50:233–8. https://iranjournals.nlai.ir/handle/123456789/805324. 22592572

[270] Biedrzycki G Wolszczak-Biedrzycka B Dorf J Michalak D Żendzian-Piotrowska M Zalewska A. The antiglycation potential of H1 receptor antagonists - in *vitro* studies in bovine serum albumin model and in silico molecular docking analyses. BioMed Pharmacother. (2024) 175:1–15. doi: 10.1016/j.biopha.2024.116632 38663107

[271] (PDF) Studying the Relationship Between Oxidative Stress Malondialdehyde and Heamatological Parameters in patients With Asthma in AL-Muthanna Province-Iraq . Available online at: https://www.researchgate.net/publication/335687716_Studying_the_Relationship_Between_Oxidative_Stress_Malondialdehyde_and_Heamatological_Parameters_in_patients_With_Asthma_in_AL-Muthanna_Province-Iraq (Accessed December 30, 2021).

[272] Peroni DG Bodini A Corradi M Coghi A Boner AL Piacentini GL. Markers of oxidative stress are increased in exhaled breath condensates of children with atopic dermatitis. Br J Dermatol. (2009) 166:839–43. doi: 10.1111/J.1365-2133.2011.10771.X 22175656

[273] Blanc F Vissers YM Adel-Patient K Rigby NM Mackie AR Gunning AP. Boiling peanut Ara h 1 results in the formation of aggregates with reduced allergenicity. Mol Nutr Food Res. (2011) 55:1887–94. doi: 10.1002/MNFR.201100251 22086730

[274] Vissers YM Iwan M Adel-Patient K Stahl Skov P Rigby NM Johnson PE. Effect of roasting on the allergenicity of major peanut allergens Ara h 1 and Ara h 2/6: the necessity of degranulation assays. Clin Exp Allergy. (2011) 41:1631–42. doi: 10.1111/J.1365-2222.2011.03830.X 21801247

[275] Maleki SJ Chung SY Champagne ET Raufman JP. The effects of roasting on the allergenic properties of peanut proteins. J Allergy Clin Immunol. (2000) 106:763–8. doi: 10.1067/MAI.2000.109620 11031348

[276] Gruber P Becker WM Hofmann T. Influence of the maillard reaction on the allergenicity of rAra h 2, a recombinant major allergen from peanut (Arachis hypogaea), its major epitopes, and peanut agglutinin. J Agric Food Chem. (2005) 53:2289–96. doi: 10.1021/JF048398W 15769170

[277] Vissers YM Blanc F Skov PS Johnson PE Rigby NM Przybylski-Nicaise L. Effect of heating and glycation on the allergenicity of 2S albumins (Ara h 2/6) from peanut. PloS One. (2011) 6:e23998. doi: 10.1371/JOURNAL.PONE.0023998 21901150 PMC3162016

[278] Iwan M Vissers YM Fiedorowicz E Kostyra H Kostyra E Savelkoul HFJ. Impact of Maillard reaction on immunoreactivity and allergenicity of the hazelnut allergen Cor a 11. J Agric Food Chem. (2011) 59:7163–71. doi: 10.1021/JF2007375 21563837

[279] Maillard reaction and enzymatic browning affect the allergenicity of Pru av 1, the major allergen from cherry (Prunus avium). Available online at: https://pubmed.ncbi.nlm.nih.gov/15186129/ (Accessed December 30, 2021). 10.1021/jf035458+ 15186129

[280] Sancho AI Rigby NM Zuidmeer L Asero R Mistrello G Amato S. The effect of thermal processing on the IgE reactivity of the non-specific lipid transfer protein from apple, Mal d 3. Allergy. (2005) 60:1262–8. doi: 10.1111/J.1398-9995.2005.00876.X 16134992

[281] Nakamura A Watanabe K Ojima T Ahn DH Saeki H. Effect of maillard reaction on allergenicity of scallop tropomyosin. J Agric Food Chem. (2005) 53:7559–64. doi: 10.1021/JF0502045 16159186

[282] Nakamura A Sasaki F Watanabe K Ojima T Ahn DH Saeki H. Changes in allergenicity and digestibility of squid tropomyosin during the Maillard reaction with ribose. J Agric Food Chem. (2006) 54:9529–34. doi: 10.1021/JF061070D 17147442

[283] Jiménez-Saiz R Belloque J Molina E López-Fandiño R. Human Immunoglobulin E (IgE) Binding to Heated and Glycated Ovalbumin and Ovomucoid before and after in Vitro Digestion. J Agric Food Chem. (2011) 59:10044–51. doi: 10.1021/JF2014638 21846147

[284] Taheri-Kafrani A Gaudin JC Rabesona H Nioi C Agarwal D Drouet M. Effects of heating and glycation of beta-lactoglobulin on its recognition by IgE of sera from cow milk allergy patients. J Agric Food Chem. (2009) 57:4974–82. doi: 10.1021/JF804038T 19489627

[285] Yieh L McEvoy CT Hoffman SW Caughey AB MacDonald KD Dukhovny D. Cost effectiveness of vitamin c supplementation for pregnant smokers to improve offspring lung function at birth and reduce childhood wheeze/asthma. J Perinatol. (2018) 38:820–7. doi: 10.1038/S41372-018-0135-6 PMC6414472 29785060

[286] Vollbracht C Raithel M Krick B Kraft K Hagel AF. Intravenous vitamin C in the treatment of allergies: an interim subgroup analysis of a long-term observational study. J Int Med Res. (2018) 46:3640–55. doi: 10.1177/0300060518777044 PMC6136002 29950123

[287] Kocyigit A Erel O Gurel MS Avci S Aktepe N. Alterations of serum selenium, zinc, copper, and iron concentrations and some related antioxidant enzyme activities in patients with cutaneous leishmaniasis. Biol Trace Elem Res. (1998) 65:271–81. doi: 10.1007/BF02789102 9892499

